# BASiCS: Bayesian Analysis of Single-Cell Sequencing Data

**DOI:** 10.1371/journal.pcbi.1004333

**Published:** 2015-06-24

**Authors:** Catalina A. Vallejos, John C. Marioni, Sylvia Richardson

**Affiliations:** 1 MRC Biostatistics Unit, Cambridge Institute of Public Health, Cambridge, United Kingdom; 2 EMBL European Bioinformatics Institute, Cambridge, United Kingdom; University of Toronto, CANADA

## Abstract

Single-cell mRNA sequencing can uncover novel cell-to-cell heterogeneity in gene expression levels in seemingly homogeneous populations of cells. However, these experiments are prone to high levels of unexplained technical noise, creating new challenges for identifying genes that show genuine heterogeneous expression within the population of cells under study. BASiCS (Bayesian Analysis of Single-Cell Sequencing data) is an integrated Bayesian hierarchical model where: (i) cell-specific normalisation constants are estimated as part of the model parameters, (ii) technical variability is quantified based on spike-in genes that are artificially introduced to each analysed cell’s lysate and (iii) the total variability of the expression counts is decomposed into technical and biological components. BASiCS also provides an intuitive detection criterion for highly (or lowly) variable genes within the population of cells under study. This is formalised by means of tail posterior probabilities associated to high (or low) biological cell-to-cell variance contributions, quantities that can be easily interpreted by users. We demonstrate our method using gene expression measurements from mouse Embryonic Stem Cells. Cross-validation and meaningful enrichment of gene ontology categories within genes classified as highly (or lowly) variable supports the efficacy of our approach.

## Author Summary

Gene expression signatures have historically been used to generate molecular fingerprints that characterise distinct tissues. Moreover, by interrogating these molecular signatures it has been possible to understand how a tissue’s function is regulated at the molecular level. However, even between cells from a seemingly homogeneous tissue sample, there exists substantial heterogeneity in gene expression levels. These differences might correspond to novel subtypes or to transient states linked, for example, to the cell cycle. Single-cell RNA-sequencing, where the transcriptomes of individual cells are profiled using next generation sequencing, provides a method for identifying genes that show more variation across cells than expected by chance, which might be characteristic of such populations. However, single-cell RNA-sequencing is subject to a high degree of technical noise, making it necessary to account for this to robustly identify such genes. To this end, we use a fully Bayesian approach that jointly models extrinsic spike-in molecules with genes from the cells of interest allowing better identification of such genes than previously described computational strategies. We validate our approach using data from mouse Embryonic Stem Cells.

## Introduction

**Fig 1:**
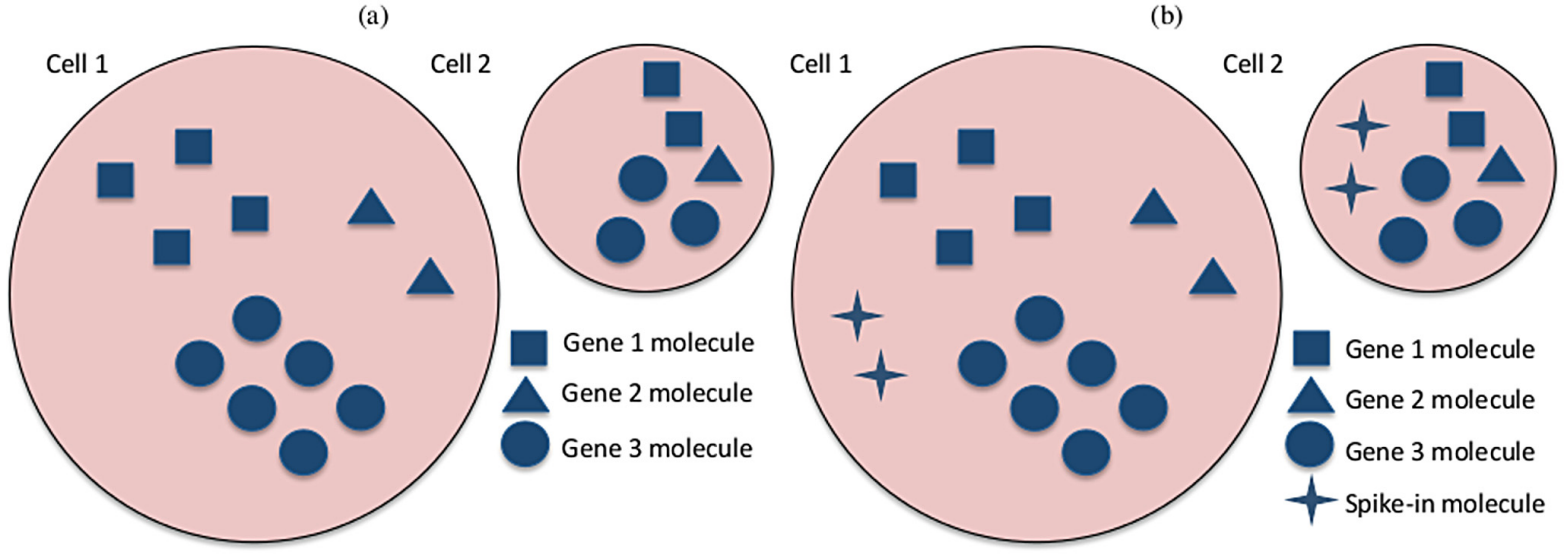
Graphical representation of gene expression in two cells from a homogeneous population but with different total mRNA content. (a) Three biological genes have the same expression rates in both cells, however cell 1 doubles cell 2 in terms of total mRNA content. As a result, the expression counts in cell 1 will be roughly twice as much as those from cell 2, for all genes. In terms of the cell-specific size factors *ϕ*_*j*_, an appropriate normalisation in this case would be e.g. *ϕ*_1_ = 2, *ϕ*_2_ = 1 (or any other values such that *ϕ*_1_/*ϕ*_2_ = 2). (b) The same cells after the addition of two molecules of a spike-in gene to each cell. Because the same number of spike-in molecules are added to each cell, the spike-in expression counts are independent of the total mRNA content of each cell. Therefore, the cell-specific size factors *ϕ*_*j*_ are not required when modelling the technical gene.

Current technology allows the analysis of gene expression with high resolution. Instead of measuring average expression levels across a bulk population, scientists can now report information at the single cell level using techniques such as single-cell RNA-sequencing (scRNA-seq) [1]. Unlike bulk experiments, scRNA-seq can uncover heterogenous gene expression patterns in seemingly homogeneous populations of cells [2], opening the door to important biological questions that remain otherwise unanswered. However, besides experimental challenges such as the isolation of single cells and parallel sequencing of multiple cDNA libraries [3], statistical analysis of single-cell level data is itself a challenge [4]. Firstly, cell-specific measurements can vary in scale due to differences in total cellular mRNA content [5]. For instance, in Fig 1(a), each gene has the same expression rate in both cells, yet the expression counts in the first cell will be roughly twice as much as those from the second cell. In the same spirit, if different sequencing depths (the number of times a single nucleotide is read during the sequencing) are applied to these cells, the scale of expression counts will also be affected. Thus, normalisation is a crucial issue in this context. Another fundamental problem for interpreting single-cell sequencing is the presence of high levels of unexplained technical noise (unrelated to sequencing depth and other amplification biases) [5]. This creates new challenges for identifying genes that show genuine biological cell-to-cell heterogeneity—beyond that induced by technical variation—and motivates the systematic inclusion of spike-in genes in single-cell experiments. Quantifying genuine heterogeneity in gene expression is an important step as it can lead to the discovery of co-expressed genes and novel cell subpopulations, among others [4][6]. Recently, the introduction of Unique Molecular Identifiers (UMI) attached to each cDNA molecule during reverse transcription has substantially reduced the levels of unexplained technical noise and eliminated the effect of sequencing depth changes and other amplification biases in single-cell experiments. Unlike most scRNA-seq datasets published to date—where expression counts likely correspond to the number of reads mapped to each gene—UMI based datasets are recorded in terms of the number of molecules, producing a meaningful scale for the expression counts. Nevertheless, our analysis of a mouse Embryonic Stem Cells (ESC) suggests that unexplained technical variability can not be completely removed by using UMIs (see Results section) and that an accurate quantification of technical variability still remains important.

Throughout, we motivate our method using UMI-based expression counts. However, the methodology described here is general and can be also extended to traditional scRNA-seq experiments (where expression counts represent the number of short reads mapped to specific genes) by modifying the interpretation of some model parameters. Typical UMI based scRNA-seq data can be represented by a *q* × *n* matrix whose entries are the number of mRNA molecules mapped to specific genes (proxy for gene expression) for each cell. More specifically, let *X*_*ij*_ be a random variable representing the expression count of a gene *i* in cell *j* (*i* = 1, …, *q*; *j* = 1, …, *n*). Thus, in a homogeneous population of cells where the true concentration of fragments from a gene *i* is *μ*_*i*_ (in a suitable unit) and where measurements are not affected by unexplained technical error, *X*_*ij*_ follows a Poisson distribution with rate *ϕ*_*j*_ *s*_*j*_ *μ*_*i*_, where *ϕ*_*j*_ adjusts the expression rate in terms of total mRNA content in cell *j* and *s*_*j*_ accounts for changes in capture efficiency across cells (for read-based expression counts, the latter also captures differences in sequencing depth and other amplification biases). Nonetheless, the Poisson model often predicts smaller variability than is observed in real datasets [7]. This so-called overdispersion is potentially linked to genes whose expression has a substantially larger biological cell-to-cell variability than would be expected in a homogeneous population of cells. However, this excess of variability may also arise from unexplained technical noise [6].

Non-biological spike-in genes (which are added to the lysis buffer and thence present at the same level in every cell) can be used to quantify technical noise (differences in capture efficiency and other unexplained sources). A typical example is the set of 92 extrinsic molecules derived by the External RNA Controls Consortium (ERCC) [8]. As the number of spike-in molecules added to each cell is known from experimental information, this provides a gold standard to which empirical measurements of spike-in genes’ expression can be compared, enabling a quantitative calibration of the technical noise. Similar strategies have also been used in the context of measurement error problems, where a validation *error free* group or gold standard measurements provide information about unknown sources of error (e.g. [9]).

Consistent with previous related literature (e.g. [5], [7]), we introduce a model based on a Poisson structure. In BASiCS (Bayesian Analysis of Single-Cell Sequencing data), a joint model of biological and spike-in genes is formulated to simultaneously quantify unexplained technical noise and cell-to-cell biological heterogeneity using the complete set of data, borrowing information between both sets of genes (spike-in and biological) through common parameters in a hierarchical structure. Additionally, BASiCS incorporates an automated normalisation method, where normalising constants are treated as model parameters. These constitute major methodological advantages over previous 3-step approaches, where first datasets are pre-normalised and secondly unexplained technical noise is estimated using only the spike-in genes, before these parameters are plugged in when modelling biological data (ignoring the uncertainty related to the technical fit).

## Materials and Methods

### The BASiCS model

**Fig 2:**
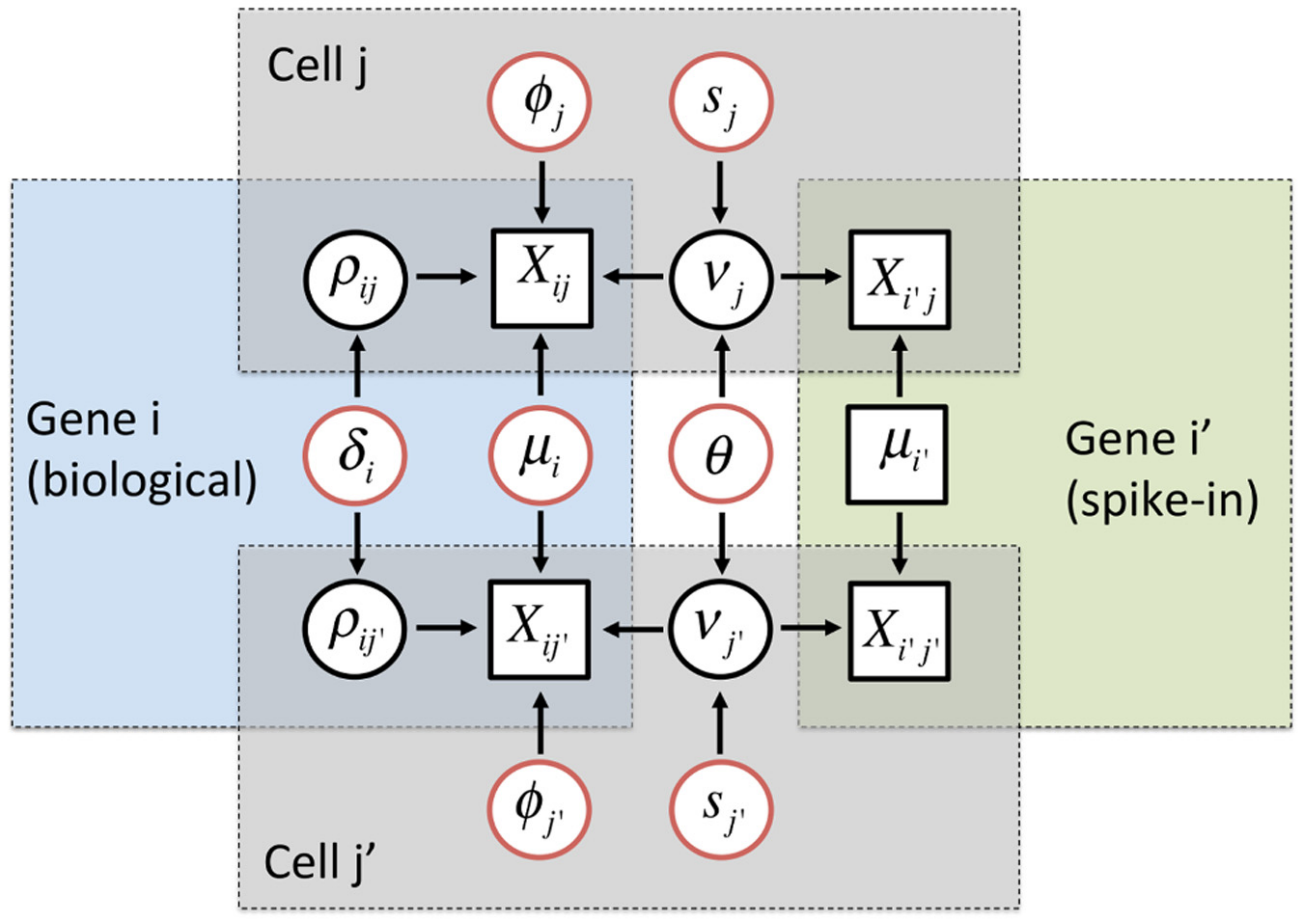
Graphical representation of the hierarchical model implemented in BASiCS. Diagram based on the expression counts of 2 genes (*i*: biological and *i*′: technical) at 2 cells (*j* and *j*′). Squared and circular nodes denote known observed quantities (observed expression counts and added number of spike-in mRNA molecules) and unknown elements, respectively. Whereas black circular nodes represent the random effects that play an intermediate role in our hierarchical structure, red circular nodes relate to unknown model parameters in the top layer of hierarchy in our model. Blue, green and grey areas highlight elements that are shared within a biological gene, technical gene or cell, respectively. BASiCS treats cell-specific normalising constants (*ϕ*_*j*_’s and *s*_*j*_’s) as model parameters, and estimates them by combining information across all genes. Unexplained technical noise is quantified via a single hyper-parameter *θ*, borrowing information across all genes and cells. Finally, BASiCS quantifies biological cell-to-cell variability via gene-specific hyper-parameters *δ*_*i*_, borrowing information across all cells.

Throughout, we analyse the expression counts of *q* genes, where *q*_0_ are expressed in the population of cells under study (biological genes) and the remaining *q*−*q*_0_ are spike-in (technical) genes. Let *X*_*ij*_ be a random variable representing the expression count of a gene *i* in cell *j* (*i* = 1, …, *q*; *j* = 1, …, *n*). Firstly, we define a model for the technical genes, whose expression counts are not affected by total cellular mRNA content (see Fig 1(b)), thus the cell-specific size factors *ϕ*_*j*_ are not required (in this case, the normalisation must only account for differences in capture efficiency via the *s*_*j*_’s). Naturally, for spike-in genes, deviations from a Poisson formulation are due only to unexplained technical variability. We assume that this unexplained technical noise depends on cell-specific characteristics and that, for a given cell, it affects the expression counts of *all genes in the same manner*. Under this assumption, unexplained technical noise can be incorporated through the following hierarchical model

$$\begin{array}{ccccc} X_{ij}|\mu_{i},\nu_{j}\overset{\text{ind}}{∼}\text{Poisson}\left(\nu_{j}\mu_{i}\right),\;\nu_{j} & | & s_{j},\theta \overset{\text{ind}}{∼}\text{Gamma}\left(1/\theta ,1/\left(s_{j}\theta \right)\right), & & \\ & & i=q_{0}+1,\ldots ,q\text{;}\;j=1,\ldots ,n\text{,} \end{array}$$ (1)

where *μ*_*i*_ represents the normalised expression rate of gene *i* in the population of cells under study and the random effect *ν*_*j*_ (with E(*ν*_*j*_∣*s*_*j*_, *θ*) = *s*_*j*_ and $\text{Var}(\nu_{j}|s_{j},\theta )=s_{j}^{2}\theta$) fluctuates around the capture efficiency normalising constant *s*_*j*_, quantifying unexplained technical noise via a single hyper-parameter *θ*, borrowing information across all genes and cells (see Fig 2). The model in Eq (1) is equivalent to a negative binomial distribution for the expression counts (like in [7]). In order to accommodate the biological genes, BASICS extends the model in Eq (1) as

$$\begin{array}{c} X_{ij}|\mu_{i},\phi_{j},\nu_{j},\rho_{ij}\overset{\text{ind}}{∼}\{\begin{array}{cc} \text{Poisson}(\phi_{j}\nu_{j}\mu_{i}\rho_{ij}), & i=1,\ldots ,q_{0}\text{,}\;j=1,\ldots ,n\text{;}\\ \text{Poisson}(\nu_{j}\mu_{i}), & i=q_{0}+1,\ldots ,q\text{,}\;j=1,\ldots ,n \end{array} \end{array}$$ (2)

$$\begin{array}{c} \text{with}\;\nu_{j}\left|s_{j},\theta \overset{\text{ind}}{∼}\text{Gamma}\left(1/\theta ,1/\left(s_{j}\theta \right)\right)\;\text{and}\;\rho_{ij}\right|\delta_{i}\overset{\text{ind}}{∼}\text{Gamma}\left(1/\delta_{i},1/\delta_{i}\right), \end{array}$$ (3)

where *ν*_*j*_’s and *ρ*_*ij*_’s are mutually independent random effects and the cell-specific size factors *ϕ*_*j*_ are introduced to normalise the biological expression counts according to differences in total mRNA content (see Fig 1(a)). As in Eq (1), the *ν*_*j*_’s capture cell-to-cell unexplained technical variability, oscillating around the capture efficiency normalising constants (*s*_*j*_) according to the strength of unexplained technical variability (*θ*). The additional random effects, *ρ*_*ij*_ (with E(ρ_*ij*_∣*δ*_*i*_) = 1 and Var(*ρ*_*ij*_∣*δ*_*i*_) = *δ*_*i*_), relate to heterogeneous expression of a gene across cells, quantifying biological cell-to-cell variability via gene-specific hyper-parameters *δ*_*i*_, borrowing information across all cells (see Fig 2). Unlike previous stepwise approaches (e.g. [5]), BASiCS treats cell-specific normalising constants (*ϕ*_*j*_’s and *s*_*j*_’s) as model parameters, and estimates them by combining information across all genes (see Fig 2), providing simultaneous inference with all other model parameters.

Here, the marginal distribution of the expression count of gene *i* in cell *j* (integrating out the random effects *ν*_*j*_’s and *ρ*_*ij*_’s) induces the same expected counts as in [5]. In fact,

$$\begin{array}{ccc} & & \text{E}\left(X_{ij}|\mu_{i},\delta_{i},\phi_{j},s_{j},\theta \right)=\phi_{j}^{I_{i}}s_{j}\mu_{i},\\ & & \text{with}\;I_{i}=1\;\text{when}\;i\leq q_{0}\;\text{and}\;I_{i}=0\;\text{otherwise.} \end{array}$$ (4)

In addition, the variance of these expression counts can be decomposed as

$$\begin{array}{c} \text{Var}\left(X_{ij}|\mu_{i},\delta_{i},\phi_{j},s_{j},\theta \right)=\phi_{j}^{I_{i}}s_{j}\mu_{i}+\theta {\left[\phi_{j}^{I_{i}}s_{j}\mu_{i}\right]}^{2}+{\text{I}}_{i}\delta_{i}\left(\theta +1\right){\left[\phi_{j}^{I_{i}}s_{j}\mu_{i}\right]}^{2}. \end{array}$$ (5)

The first term in Eq (5) is the biological baseline variance—based on a Poisson($\phi_{j}^{I_{i}}s_{j}\mu_{i}$) model. The second component represents the variance inflation due to unexplained technical variability and the final term is linked to biological cell-to-cell heterogeneity. The decomposition in Eq (5) is similar (as a function of the expected counts) to those proposed in [5] and [7], which have been validated empirically.

### BASiCS: detection of highly and lowly variable genes

Intuitively, highly variable genes (HVG) are those for which a large fraction of the total expression variability is explained by a biological cell-to-cell heterogeneity component. Here, we characterise highly variable genes as those for which

$$\begin{array}{ccc} \sigma_{i}\equiv \frac{\delta_{i}\left(\theta +1\right)}{{\left[{\left(\phi s\right)}^{*}\mu_{i}\right]}^{-1}+\theta +\delta_{i}\left(\theta +1\right)}>\gamma_{{}_{H}}, & & \\ \text{where}\;{\left(\phi s\right)}^{*}=\underset{j\in \{1,\ldots ,n\}}{\text{median}}\{\phi_{j}s_{j}\}, & \end{array}$$ (6)

i.e. when the proportion of the total variability of the expression counts of gene *i* in a reference cell (derived from Eq (5), replacing *ϕ*_*j*_ *s*_*j*_ by (*ϕs*)^*^ in order to represent a *typical cell* within the analysed sample) that is explained by biological cell-to-cell heterogeneity exceeds a variance contribution threshold *γ*_*H*_. In other words, we characterise as HVG those whose biological cell-to-cell heterogeneity component explains *γ*_*H*_ × 100% of the total variability (in a typical cell). The latter criterion induces contours in terms of *δ*_*i*_, which are given by

$$\begin{array}{c} \delta_{i}>[\frac{\gamma_{{}_{H}}}{1-\gamma_{{}_{H}}}][\frac{{\left({\left(\phi s\right)}^{*}\mu_{i}\right)}^{-1}+\theta }{1+\theta }]. \end{array}$$ (7)

Naturally, the contour in Eq (7) is an increasing function of *γ*_*H*_. Additionally, it is a decreasing function of the normalised expression rate *μ*_*i*_, which is a welcome feature (previous studies have shown evidence of lower levels of biological cell-to-cell heterogeneity in highly expressed genes [5]).

BASiCS quantifies the evidence in favour of a gene being highly variable in terms of the upper tail posterior probabilities (associated to high biological cell-to-cell heterogeneity components) and labels as HVG those genes such that (for a given evidence threshold *α*_*H*_)

$$\begin{array}{c} \pi_{i}^{H}\left(\gamma_{{}_{H}}\right)=\text{P}(\sigma_{i}>\gamma_{{}_{H}}|\left\{x_{ij}:i=1,\ldots ,q,j=1,\ldots ,n\right\})>\alpha_{{}_{H}}, \end{array}$$ (8)

i.e. when such evidence is strong. Analogously, lowly variable genes (LVG) would be those for which

$$\begin{array}{c} \pi_{i}^{L}\left(\gamma_{{}_{L}}\right)=\text{P}(\sigma_{i}<\gamma_{{}_{L}}|\left\{x_{ij}:i=1,\ldots ,q,j=1,\ldots ,n\right\})>\alpha_{{}_{L}}, \end{array}$$ (9)

for a given variance contribution threshold *γ*_*L*_ and an evidence threshold *α*_*L*_. Estimates of these quantities can be easily computed based on a posterior sample of the model parameters, requiring minimal computational effort (other criteria, such as Bayes Factors, usually require intensive calculations [10]). Tail posterior probabilities have also been used in the context of differential expression for microarray experiments [11], providing richer and more interpretable output than standard hypothesis testing procedures.

Our method for detecting highly (and lowly) variable genes requires the choice of variance contribution thresholds *γ*_*H*_ and *γ*_*L*_ as well as evidence thresholds *α*_*H*_ and *α*_*L*_. If there is biological motivation behind particular values of *γ*_*H*_ or *γ*_*L*_, these values can be fixed prior to the analysis. However, *α*_*H*_ and *α*_*L*_ have a technical role, quantifying the uncertainty associated with the detection of HVG and LVG. For fixed values of *γ*_*H*_ and *γ*_*L*_, we can choose optimal values for *α*_*H*_ and *α*_*L*_ as those where the expected false discovery rate (EFDR) and expected false negative rate (EFNR) coincide. For the rule in Eq (8), these quantities are defined as in [12] and respectively given by

$$\begin{array}{ccc} {\text{EFDR}}_{\alpha_{{}_{H}}}=\frac{\sum_{i=1}^{q_{0}}\left(1-\pi_{i}^{H}\left(\gamma_{{}_{H}}\right)\right)I\left(\pi_{i}^{H}\left(\gamma_{{}_{H}}\right)>\alpha_{{}_{H}}\right)}{\sum_{i=1}^{q_{0}}I\left(\pi_{i}^{H}\left(\gamma_{{}_{H}}\right)>\alpha_{{}_{H}}\right)}\;\text{and} & & \\ {\text{EFNR}}_{\alpha_{{}_{H}}}=\frac{\sum_{i=1}^{q_{0}}\pi_{i}^{H}\left(\gamma_{{}_{H}}\right)I\left(\pi_{i}^{H}\left(\gamma_{{}_{H}}\right)\leq \alpha_{{}_{H}}\right)}{\sum_{i=1}^{q_{0}}I\left(\pi_{i}^{H}\left(\gamma_{{}_{H}}\right)\leq \alpha_{{}_{H}}\right)}. & \end{array}$$ (10)

where *I(A)* = 1 if *A* is true, 0 otherwise. Equivalent expressions can be determined for Eq (9), replacing $\pi_{i}^{H}(\gamma_{H})$ and *α*_*H*_ by $\pi_{i}^{L}(\gamma_{L})$ and *α*_*L*_, respectively. Alternatively, if there is no clear pre-determined choice for *γ*_*H*_ and *γ*_*L*_, choosing a specific common value for the EFDR and the EFNR (e.g. EFDR = EFNR = 10%) can define optimal values for *α*_*H*_ and *α*_*L*_ as well as for *γ*_*H*_ and *γ*_*L*_.

Beyond the choice of particular thresholds for the detection of highly and lowly variable genes, a key advantage of our method is the generation of a natural ranking of the genes in terms of the percentage of variance explained by the biological cell-to-cell heterogeneity component (*σ*_*i*_). For particular threshold choices, our method classifies as highly variable those genes for which *σ*_*i*_ is high (above the variance contribution threshold *γ*_*H*_) and where there is strong evidence to support this fact (the probability of {*σ*_*i*_ > *γ*_*H*_} is above the evidence threshold *α*_*H*_). As a result, BASiCS aims to identify key drivers of cell-to-cell heterogeneity rather than complete enumeration. Our analysis does not imply that all genes located below these thresholds have stable expression among the analysed cells.

### BASiCS: identifiability

Without additional assumptions, the parameters of the model presented in Eq (2) and Eq (3) cannot be identified. However, the cell-specific capture efficiency normalising terms *s*_*j*_’s can be identified if we assume that *μ*_*q*_0_+1_, …, *μ*_*q*_ are known. This is not a limitation, because the true concentration of the spike-in genes added to each cell are known from experimental information. In addition, *δ*_*i*_’s (quantifying gene-specific biological cell-to-cell heterogeneity) and *θ* (quantifying unexplained technical variability) can be identified via the variance of the biological and technical expression counts. Nonetheless, the scale of the *ϕ*_*j*_’s (cell-specific mRNA content normalisation) is arbitrary because *μ*_1_, …, *μ*_*q*_0__ are unknown. A simple solution is to impose the restriction $n^{-1}\sum_{j=1}^{n}\phi_{j}=\phi_{0}$, which can be achieved by reparameterising the model in terms of *κ*_1_, …, *κ*_*n*_ with

$$\begin{array}{c} \phi_{j}=\phi_{0}\frac{\;e^{\kappa_{j}}}{\sum_{j=1}\;e^{\kappa_{j}}},j=1,\ldots ,n\;\kappa_{1}=0. \end{array}$$ (11)

Although this restriction imposes an arbitrary scale to the *ϕ*_*j*_’s, this does not affect inference about *relative differences* between the *μ*_*i*_’s, nor the *δ*_*i*_’s. Therefore, standard analyses, such as the detection of highly variable genes or differential expression, are not affected by particular values of *ϕ*_0_. For simplicity, we recommend *ϕ*_0_ = *n* (this value will be used hereafter in this article).

### BASiCS: prior specification and implementation of posterior inference

We assume prior independence between all model parameters, using a flat *non-informative* prior for the normalised expression rates *μ*_1_, …, *μ*_*q*_0__ and proper *informative* prior distributions for all other model parameters. Under this prior, Bayesian inference is implemented using an Adaptive Metropolis (AM) within Gibbs Sampling (GS) algorithm [13]. This algorithm was implemented using a combination of C++ and R via the Rcpp library [14]. An R package has been prepared and is available at: https://github.com/catavallejos/BASiCS

More details about the prior specification and the implementation of posterior inference can be found in S1 Text and S2 Text, respectively. Information regarding the computational cost of our method is displayed in S7 Text.

### Alternative method presented in Brennecke et al (2013)

**Fig 3:**
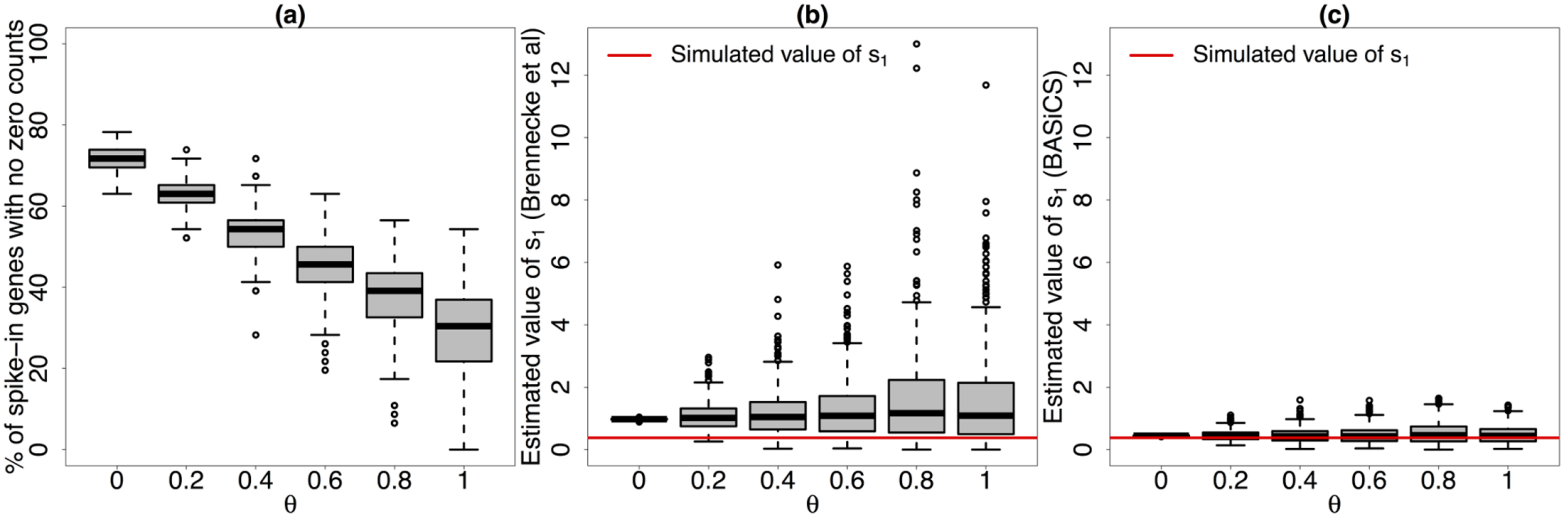
Simulated performance of *s*_*j*_’s estimates (method described in [5] and BASiCS). Based on 400 simulated datasets from the model implemented in BASiCS with the same structure as in the mouse ESC dataset (simulated parameter values defined as posterior medians of the original model fit) and 6 different values for *θ*. (a) percentage of the simulated spike-in genes (out of 46) without zero counts (i.e. those that can be used when calculating the estimator proposed in [5]) for different simulated values of *θ*. (b) and (c) estimates of *s*_1_ (first cell) across all simulated datasets for different simulated values of *θ* using the method described in [5] and BASiCS (posterior medians), respectively. As the strength of unexplained technical noise increases (larger values of *θ*), estimates obtained using the approach described in [5] become highly unstable (we illustrated this using the first simulated cell, but the same conclusion can be obtained based on any other cell). This is due to a larger proportion of zeros among the simulated expression counts, i.e. less spike-in genes can be used when estimating *s*_1_. In contrast, the stability of the BASiCS estimates is not substantially affected by the strength of unexplained technical noise.

Here, we briefly discuss the 3-step method described in [5] to analyse scRNA-seq data and to detect HVG in the population of cells under study (notably, BASiCS not only provides a method for HVG detection, but LVG can also be identified). This method pre-normalises the expression counts using the method available in DESeq [15], calculating two separate sets of normalising constants as:

$$\begin{array}{ccc} & & \omega_{j}^{B}={\text{median}}_{i=1,\ldots ,q_{0}}\{\frac{x_{ij}}{{\left(\prod_{j=1}^{n}x_{ij}\right)}^{1/n}}\}\;\text{and}\\ & & \omega_{j}^{T}={\text{median}}_{i=q_{0}+1,\ldots ,q}\{\frac{x_{ij}}{{\left(\prod_{j=1}^{n}x_{ij}\right)}^{1/n}}\},j=1,\ldots ,n \end{array}$$ (12)

for biological and technical genes, respectively (in Eq (12), *x*_*ij*_ represents the observed counts of a gene *i* in cell *j*). In terms of our notation, $\omega_{j}^{B}$ and $\omega_{j}^{T}$ play the role of *ϕ*_*j*_ *s*_*j*_ and *s*_*j*_, respectively. Based on point estimates of these quantities, normalised expression counts are then computed as $x_{ij}^{*}=x_{ij}/\omega_{j}^{B}$ and $x_{ij}^{*}=x_{ij}/\omega_{j}^{T}$ for biological and technical genes, respectively. When a large number of genes is being analysed, the variance associated to estimators in Eq (12) is negligible. However, the expressions in Eq (12) are undefined if one or more of the expression counts of any analysed gene are equal to zero (the geometric means in the denominators are equal to zero). A common solution is to exclude those genes with zero counts from the normalisation calculations (but not from any other downstream analysis). As a result, these estimators become highly unstable, especially for strong levels of technical noise (where a high proportion of zero counts is typically observed). This is illustrated in Fig 3 (see panels (a) and (b)), where we simulated data using the same structure as the mouse ESC dataset analysed in the Results section, using the model implemented in BASiCS and a range of values for *θ* (including *θ* = 0, where there is no unexplained technical noise). Fig 3(b) also shows that the stepwise approach proposed in [5] does not recover the correct scale for the *s*_*j*_’s (not surprising as their method was not designed to do so). In contrast, Fig 3(c) shows the superior performance of our approach. This is not surprising because: (i) our estimates used the actual expression rates of the spike-in genes (given by the number of mRNA ERCC molecules added to the lysis buffer of each cell), instead of their empirical counterparts (recovering a correct and meaningful scale for the *s*_*j*_’s) and (ii) we combined information from all genes (biological and technical) without having to exclude genes where one or more cell-specific counts were equal to zero.

Using the pre-normalised expression counts, [5] proposes a HVG detection method based on the relationship between gene-specific sample means and the corresponding coefficients of variation. An initial fit of this relationship is made using only the spike-in genes (where heterogeneous expression is only due to a technical component), quantifying the effect of unexplained technical variability. The output of this technical fit is then plugged in when modelling biological data, characterising as HVG those whose expression variability substantially exceeds what would be expected due to technical variability (i.e. the level predicted by the technical fit)—ignoring the uncertainty associated to the technical fit.

## Results

### Motivating data: mouse ESC presented in Islam et al (2014)

To illustrate BASiCS we consider scRNA-seq data for 7,941 genes (7,895 biological and 46 ERCC spikes) from 41 mouse ESCs. This corresponds to a subset of the dataset presented in [16], generated by discarding those genes with total count (across all cells) below 41 (i.e. where the counts are, on average, less than 1 molecule per cell). By doing this, we exclude genes with very low expression rates, which have less biological relevance. As illustrated in [16], the use of UMIs (attached to each cDNA molecule during reverse transcription) reduces the strength of technical noise. Nevertheless, our analysis suggests that unexplained technical variability has not been completely removed by this technology (see discussion below). S3 Text describes the input parameters used for the implementation (including prior hyper-parameters values). The data and code used for the analysis are provided in S1 Data.

### Normalisation

**Fig 4:**
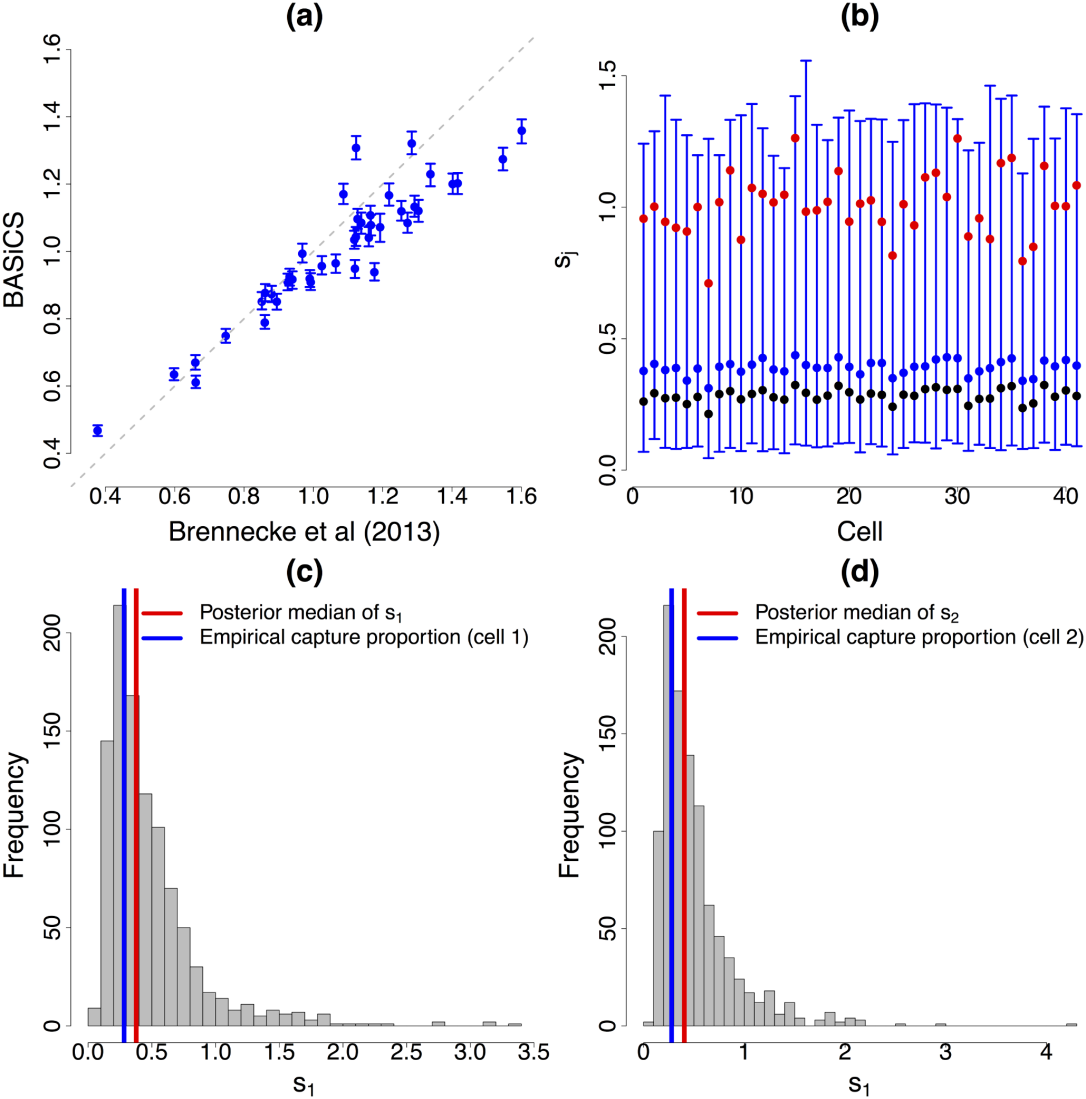
Normalisation. (a) and (b): for each of the 41 mouse ESCs, vertical lines represent the 95% high posterior density interval (blue dot located at the posterior median) of cell-specific normalising constants *ϕ*_*j*_ (cellular mRNA content) and *s*_*j*_ (interpreted in terms of capture and reverse transcription efficiency for UMI counts), respectively. While BASiCS suggests substantial heterogeneity in the total amount of molecules per cell (*ϕ*_*j*_), the scale of the technical counts remains stable among cells (*s*_*j*_). This is expected when using UMI protocols, where counts should not be affected by sequencing depth and other amplification biases. Red dots are the values estimated by the stepwise method described in [5]. There is a good agreement of the methods in terms of cellular mRNA content (*ϕ*_*j*_), but the estimations of *s*_*j*_ according to [5] suggest stronger differences than what is expected when using UMI protocols. In (b), black dots represent the proportion of total spike-in molecules captured in each cell. Our estimations of the *s*_*j*_’s are in better agreement with these empirical measurements (suggesting BASiCS infers a more adequate reverse transcription efficiency level). (c) and (d) histogram of a Markov Chain Monte Carlo sample from *s*_1_ and *s*_2_, respectively. These posterior distributions are highly skewed and thence the posterior modes are a closer match to the empirical capture proportions than the corresponding posterior medians.

Fig 4 summarises posterior inference for the cell-specific normalising terms *ϕ*_*j*_’s and *s*_*j*_’s. Panel (a) suggests there is a substantial heterogeneity in the total mRNA content per cell (*ϕ*_*j*_) and a relatively good correspondence between our estimates and the ones produced by the method in [5]. In the context of UMI datasets, the *s*_*j*_’s can be understood as a measure of changes in capture efficiency. In the ideal case, all the *s*_*j*_ coefficients should approach 1 (i.e. all gene molecules are captured). Instead, in the case of the analysed mouse ESCs, the posterior medians of the *s*_*j*_’s vary between 0.31 and 0.44 across cells (see panel (b)), suggesting that part of the original molecules are lost throughout the experiment (this is particularly critical for lowly expressed genes, as they might not be captured at all). As shown in panel (b), the BASiCS estimations of the *s*_*j*_’s show good concordance with the empirical proportions of total spike-in molecules captured in each cell. The small scale difference between the posterior medians of the *s*_*j*_’s and these empirical proportions is due to a highly skewed posterior distribution of the *s*_*j*_’s; however posterior modes closely match these values (see panels (c) and (d)). Panel (b) also shows a strong discrepancy between the methods when estimating the *s*_*j*_’s. Our method suggests that the scale of the technical counts does not substantially vary among cells, which is more reasonable when analysing UMI-based counts. Finally, an important feature of our method is a direct quantification of the uncertainty related to estimation of all normalising constants *ϕ*_*j*_’s and *s*_*j*_’s (by means of high posterior density intervals), an element that was ignored in [5].

### Variance decomposition and detection of HVG/LVG

**Fig 5:**
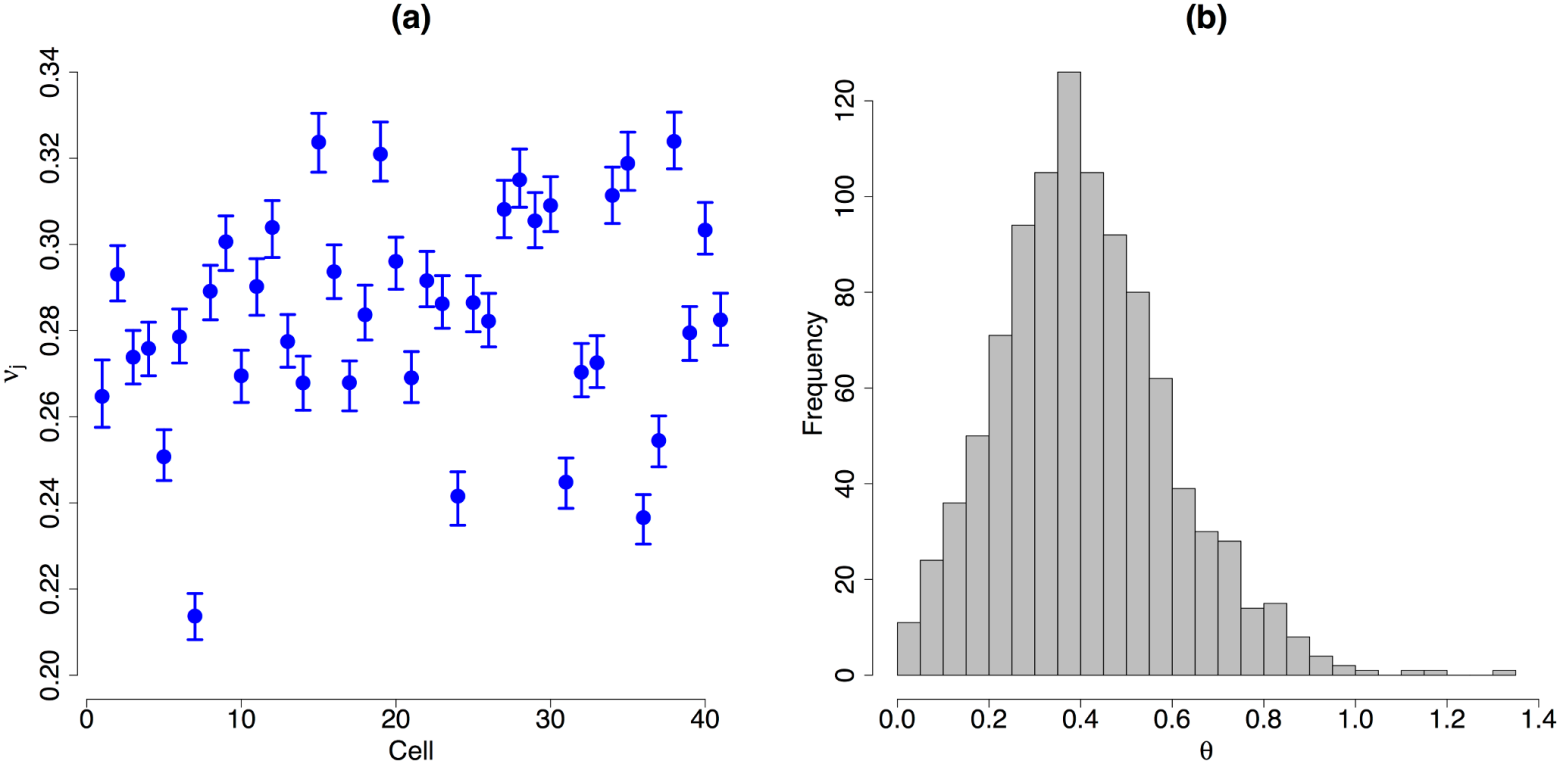
Cell-specific random effects linked to unexplained technical variability. (a): for each of the 41 mouse ES cells, vertical lines represent the 95% high posterior density interval (blue dot located at the posterior median) of the random effects related to unexplained technical cell-to-cell variability (*ν*_*j*_). (b): histogram of a Markov Chain Monte Carlo sample from *θ*. Posterior inference strongly suggests the presence of unexplained technical noise in gene expression measurements. In fact, the posterior distribution of *θ* is concentrated away from zero and—even though the posterior distributions of the *s*_*j*_’s are highly homogeneous across cells (see Fig 4(b))—there is a strong heterogeneity among the posterior distributions of the *ν*_*j*_’s (evidenced by non-overlapping 95% high posterior density intervals).

**Fig 6:**
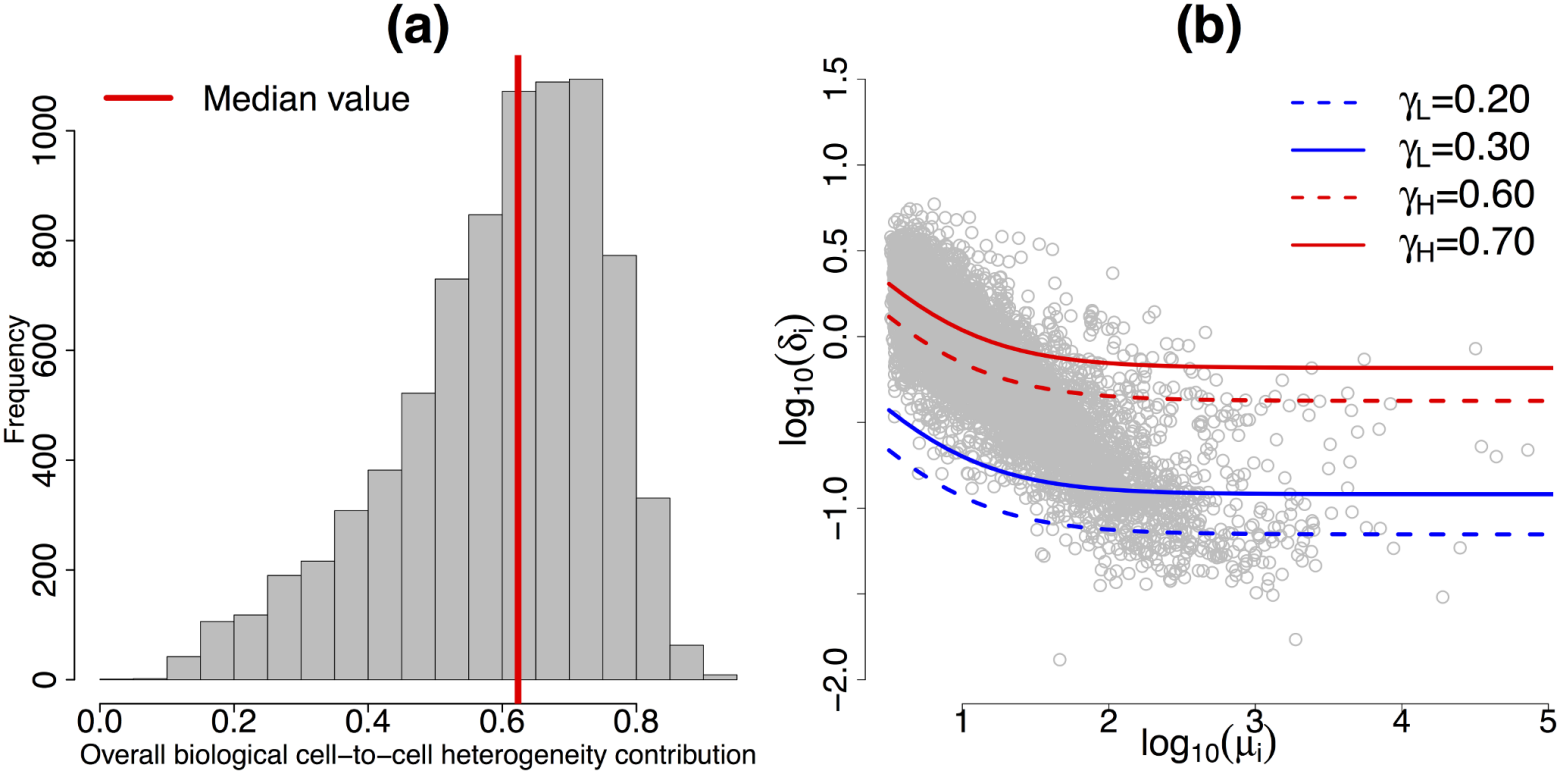
Biological cell-to-cell heterogeneity. (a): Histogram of the posterior medians of gene-specific biological cell-to-cell heterogeneity variance contributions *σ*_*i*_ (defined in Eq (6)) across the 7,895 biological genes. (b): For each of the 7,895 biological genes, posterior medians of biological cell-to-cell heterogeneity term *δ*_*i*_ (log scale) against posterior medians of expression level *μ*_*i*_ (log scale). Red lines represent the contours in Eq (7), related to HVG (log scale) at different levels of the variance contribution threshold *γ*_*H*_. Blue lines represent the equivalent contours linked to LVG at different levels of the variance contribution threshold *γ*_*L*_. These contours were estimated based on posterior medians of *ϕ*_*j*_’s, *s*_*j*_’s and *θ*.

Despite the use of UMIs, posterior inference strongly suggests the presence of unexplained technical noise in gene expression measurements (see Fig 5). In fact, the posterior distribution of the unexplained technical variability parameter *θ* is concentrated away from zero (see panel (b)). In addition, even though the posterior distribution of the cell-specific normalising terms *s*_*j*_ is homogeneous across cells, panel (a) shows substantial differences among the cell-specific random effects (*ν*_*j*_). Overall—across all genes—the unexplained technical component explains approximately 28% of the total variability of expression counts in a typical cell. The data also exhibit strong evidence of biological cell-to-cell heterogeneity. In fact, in the case of the analysed mouse ESC dataset, the posterior median of *σ*_*i*_ (defined in Eq (6)) is above 62% for 50% of the 7,895 biological genes (see Fig 6(a)). In addition, Fig 6(b) shows a strong relationship between the biological cell-to-cell heterogeneity (*δ*_*i*_) and the gene-specific expression rates (*μ*_*i*_) which is coherent with the contours in Eq (7) that are decreasing functions of *μ*_*i*_.

**Fig 7:**
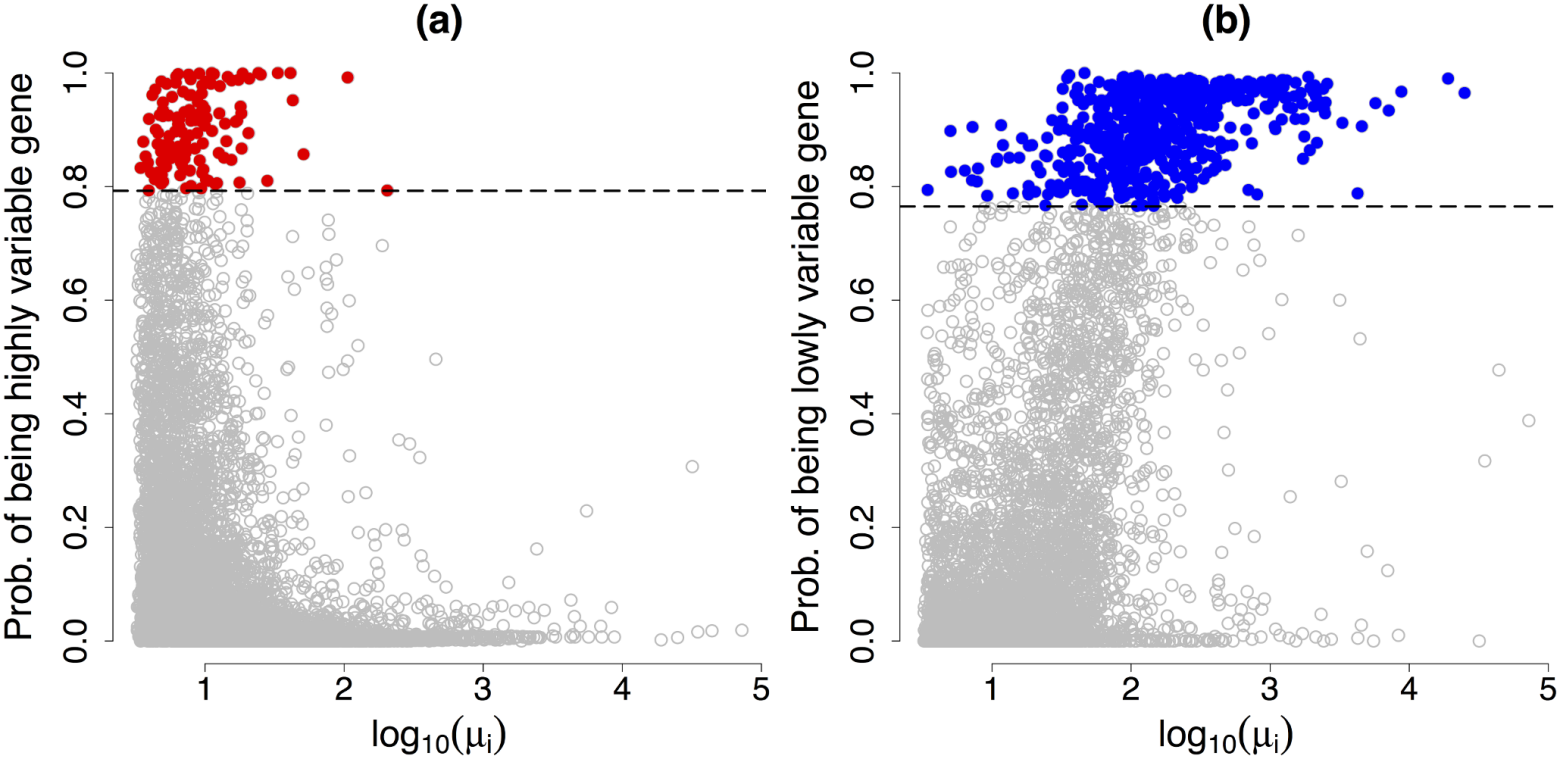
HVG and LVG detection. (a) and (b): for each of the 7,895 biological genes, gene-specific expression rate *μ*_*i*_ (log scale) against the probability of being HVG ($\pi_{i}^{H}(\gamma_{H})$) and the probability of being LVG ($\pi_{i}^{L}(\gamma_{L})$), respectively. Setting the EFDR and the EFNR equal to 10%, the corresponding variance contribution thresholds are *γ*_*H*_ = 79% and *γ*_*L*_ = 41%. Black dashed lines located at optimal (i.e. when EFDR and EFNR coincide) evidence thresholds *α*_*H*_ = 0.7925 and *α*_*L*_ = 0.7650, respectively. The 133 and 589 genes classified as HVG and LVG are highlighted in red and blue, respectively.

In practice, we define variance contribution thresholds (*γ*_*H*_ and *γ*_*L*_) and evidence thresholds (*α*_*H*_ and *α*_*L*_) for the detection of HVG and LVG by setting the EFDR and the EFNR (defined as in Eq (10)) equal to 10% (see Table S1 in S4 Text). Using this rule, we obtain *γ*_*H*_ = 0.79, *γ*_*L*_ = 0.41 (with corresponding evidence thresholds *α*_*H*_ = 0.7925, *α*_*L*_ = 0.7650). Therefore, we label as highly variable those genes for which there is strong evidence of a biological cell-to-cell heterogeneity component that explains more than 79% of the total expression variability. Similarly, we set *γ*_*L*_ = 0.41, thus defining as LVG those with strong evidence that the biological cell-to-cell heterogeneity explains less than 41% of the total expression variability. Posterior estimates of the detection probabilities associated to each gene are displayed in Fig 7. While LVG are typically associated with large expression rates, the expression rates of HVG are concentrated in a lower range. With these variance contributions and evidence thresholds, we detect 133 HVG and 589 LVG (highlighted in Fig 7, panels (a) and (b), respectively).

### Biological interpretation of HVG and LVG

**Fig 8:**
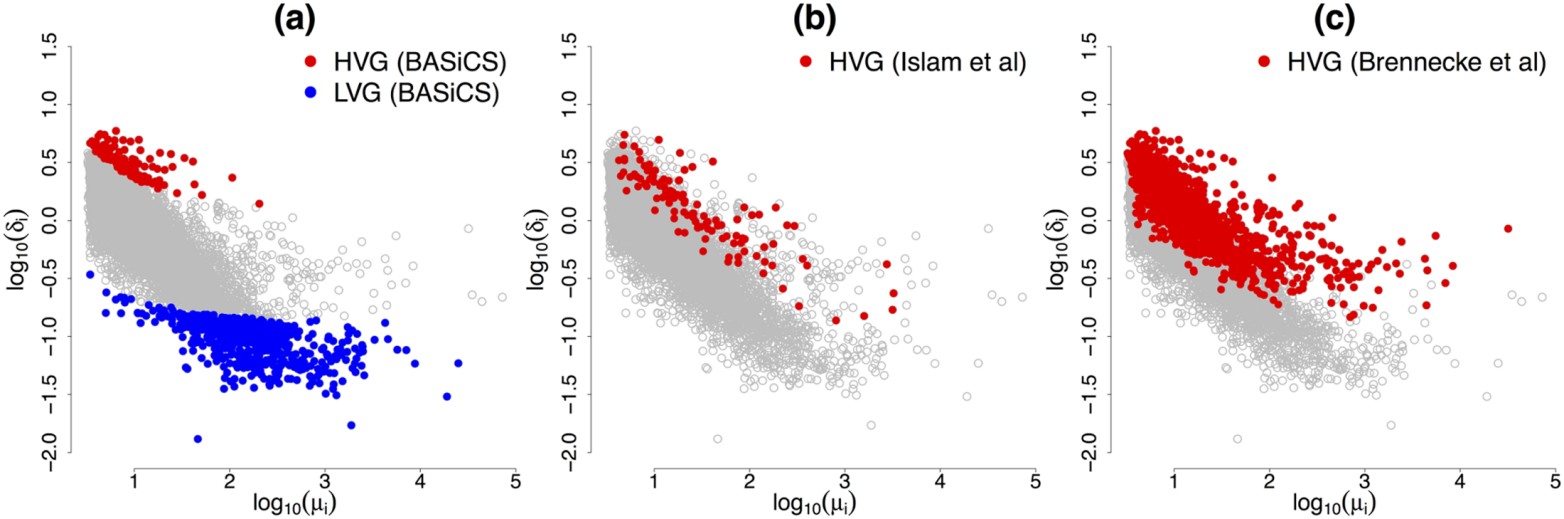
Comparison of HVG detection among different methods. For each of the 7,895 biological genes, posterior medians of biological cell-to-cell heterogeneity term *δ*_*i*_ (log scale) against posterior medians of expression level *μ*_*i*_ (log scale). While the methods described in [16] and [5] only provide a characterisation of HVG, BASiCS is able to detect those genes whose expression rates are stable among cells.

Among the 133 genes classified as HVG, there is an enrichment of genes related to cell differentiation (see Table S2 in S5 Text). These HVG include (posterior medians of *σ*_*i*_ are shown in parenthesis) *Dppa3* (85.1%) for which [17] previously showed heterogeneous expression in mouse ES cells via *in situ* hybridisation. Other genes for which [17] found heterogeneous expression did not pass our criteria, yet we estimate a substantial component of biological cell-to-cell heterogeneity associated to most of them: *Esrrb* (79.7%), *Zfp42* (75.9%), *Krt8* (67.8%), *Nanog* (66.4%), *Atf4* (64.6%), *Whsc2* (56.3%), *Rest* (48.7%), *Fscn1* (47.5%) and *Pa2g4* (27.5%). In particular, some of these genes would be classified as HVG if a slightly less conservative EFDR and EFNR threshold was adopted. Our results are more conservative than those according to the method described in [5] (see Fig 8(a)), where 1,363 genes were labelled as HVG. This is not surprising as their method suggests stronger heterogeneity among the cell-specific normalising constants *s*_*j*_’s, potentially inducing spurious heterogeneous expression in genes that remain otherwise stable. In addition, as shown in Fig 8(b), there is a relatively good correspondence between our results and the list of HVG published by [16] (their heuristic method classified as HVG those with substantially larger expression variability than would be predicted by a Poisson model, arguing that the need of normalisation and quantification of unexplained technical noise is removed by the use of UMIs). There are 23 genes classified as HVG by both methods (also detected by [5]), including e.g. *Sprr2b* (91.6%), *Dqx1* (90.9%) *Ccdc48* (90.9%), *Mreg* (89.1%) and *Fst* (88.1%). Several of the genes presented as HVG by [16] but not by us are borderline according to our criteria and exhibit a substantial, yet less predominant, intra-tissue heterogeneity (the posterior medians of *σ*_*i*_ are above 68% for 75% of them). For example, *Lefty1* exhibits a heterogeneous pattern of expression, which BASiCS reflects by estimating *σ*_*i*_ = 79.4% (yet the data does not provide enough evidence to conclude that *more than* *γ*_*H*_ = 79% of the expression variability of *Lefty1* can be attributed to a biological cell-to-cell heterogeneity component). Nonetheless, other genes identified as highly variable by [16], such as *Gapdh* (35.7%), are far from being labelled as HVG by our method ($\pi_{i}^{H}(0.79)=0.009$). The latter is more reasonable, in view of the extensive use of *Gapdh* as a reference gene in mouse ESCs [18].

The enrichment of lowly and mildly expressed genes within those highlighted as highly variable is not an artefact of our method and relates to the characteristics of the analysed mouse ESC dataset. In fact, the analysed sample includes cells from a fairly homogeneous population of cells and highly expressed genes are mostly related to key processes that are common to all cells, acting as *housekeeping genes*. To validate this, we analysed the dataset described in [19], which contains 3,005 samples from a highly heterogeneous population of cells. In such a setting, our analysis reveals that BASiCS is capable of detecting highly variable genes across the whole range of expression levels (see S8 Text).

In terms of LVG, neither [5] nor [16] can be employed. Our results are validated by a strong enrichment of genes related to core cellular processes such as translation and translational elongation (see Table S2 in S5 Text). In particular, we include *Eif5b* (12.7%) which has been previously shown to have homogeneous expression in mouse ESCs [17]. Our list of LVG also includes e.g.: *Mir466d* (4.0%), *Hsp90ab1* (5.8%), *Gm6251* (11.4%), *Zfp207* (13%) and *Arpc1b* (14.0%). *Gapdh* is not labelled as LVG, however the posterior distribution of its associated *σ*_*i*_ is heavily skewed towards small values and it would be included in the LVG list if we used a slightly higher EFDR and EFNR threshold.

### Cross-validation

**Fig 9:**
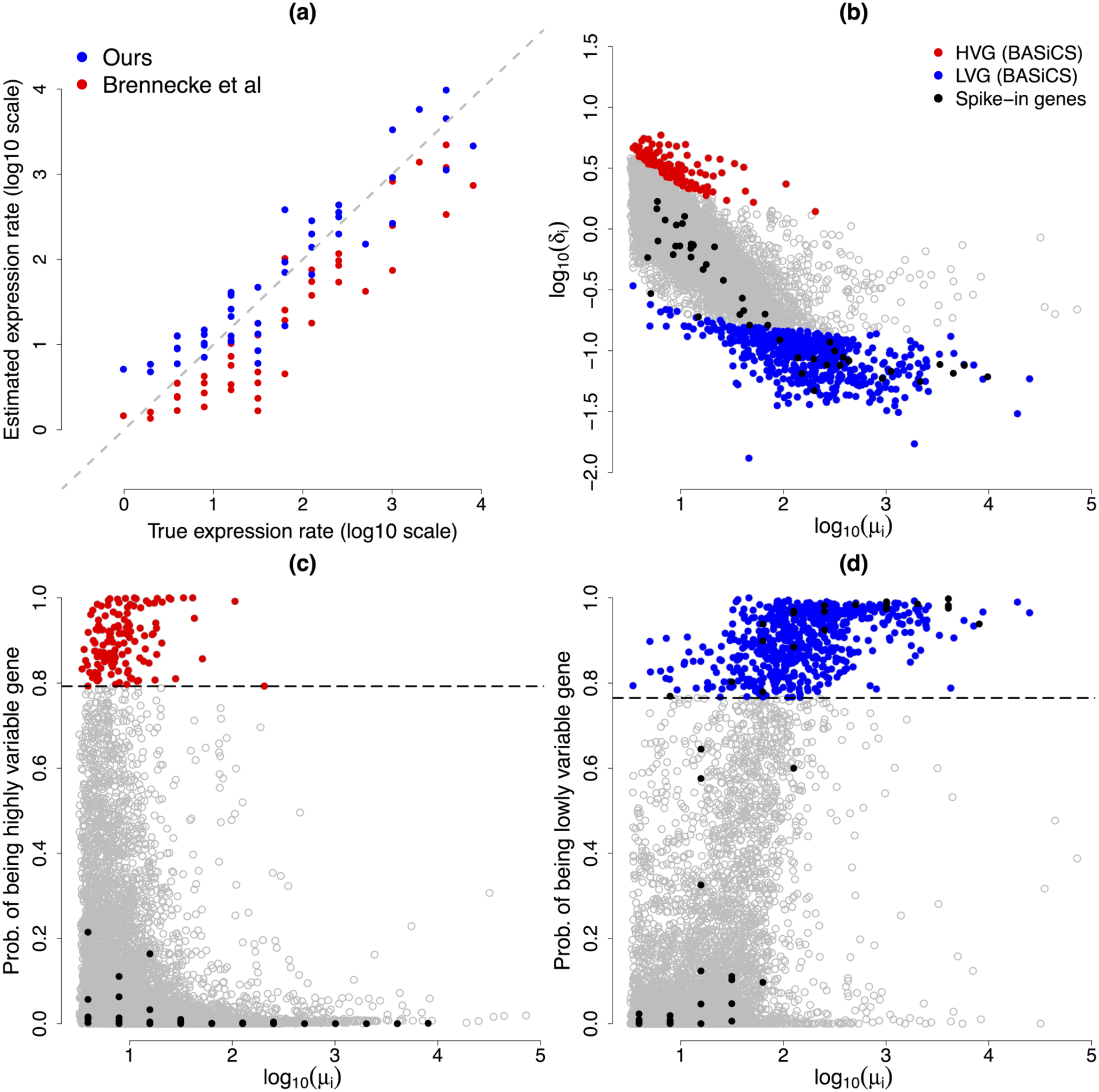
Cross-validation experiment. (a) true versus estimated normalised expression level *μ*_*i*_ for each of the 46 ERCC spike-in genes (log-scale). Our estimations are gathered around the true values, except for lowly expressed technical genes, where the experiment suggests a small positive bias. Estimations according to [5] are highly correlated with the true values, however the true scale has not been recovered (not surprising as their method is not designed to estimate the right scale of the *s*_*j*_’s, see Fig 3). (b): Fig 8(a) superimposing (in black) estimated values for each of the 46 ERCC spike-in genes. (c) and (d): panels (c) and (d) of Fig 7 superposing (in black) estimated probabilities associated to each of the 46 ERCC spike-in genes. As expected, none of the spike-in genes were detected as HVG by our criteria. On the other hand, there is strong evidence in favour of being LVG for 21 of the technical genes (and some others are borderline according to our conservative criteria).

As well as enabling estimation of the degree of unobserved technical noise, the spike-in genes can also be used to validate our method. We performed a *cross-validation*-type procedure where, for each of the 46 ERCC spike-in genes in turn, we modified the dataset by treating the selected technical gene as if it were a biological one. As the number of added mRNA molecules of these technical genes is known from experimental information, this experiment allows an assessment of our estimates of gene-specific normalised expression levels (*μ*_*i*_). As shown in Fig 9(a), our estimates are gathered around the true values, except for lowly expressed technical genes, where the experiment suggests a small positive bias. Estimates according to [5] are highly correlated with the true values, however the true scale has not been recovered (not surprising as their method is not designed to estimate the right scale of the *s*_*j*_’s, see Fig 3). In addition, we can use this analysis to validate posterior inference on the cell-to-cell biological heterogeneity components (*δ*_*i*_) and our detection criteria for HVG and LVG (see Fig 9, panels (b), (c) and (d)). As expected, none of the spike-in genes were detected as HVG by our criteria. On the other hand, there is strong evidence that 21 (out of 46) spike-in genes fall in the LVG category, with some others just failing to overcome our conservative criteria.

## Discussion

Single-cell measurements of gene expression can expose heterogeneous behaviour within seemingly homogeneous populations of cells [2]. BASiCS incorporates an integrated normalisation method where cell-specific normalising constants are estimated as model parameters. In particular, we normalise expression counts according to the mRNA content of each cell. These so-called size factors are biologically important since they can partially reflect cell cycle stage (cells tend to contain more mRNA molecules in later stages of the cell cycle [20]). To demonstrate this idea, we analysed the mouse ESC dataset described in [20], where the cell cycle stage of the analysed cells was recorded. BASiCS estimates substantially larger mRNA content for those cells captured during G2 and M phases (with respect to those in earlier stages of the cell cycle). A summary of this analysis is displayed in S6 Text.

Additionally, our joint model of biological and spike-in genes allows biological cell-to-cell variability to be teased apart from other technical sources of variability as well as facilitating the generation of a calibrated decision rule, based on easily interpretable posterior probabilities, for selecting highly or lowly variable genes in the population of cells under study. Such information can uncover sub-populations of cells with distinct patterns of gene expression as well as producing a natural ranking of genes according to their biologically variability.

Among others, future extensions of BASiCS include the implementation of differential expression analyses. BASiCS also provides a basis to build more complex downstream analyses including cluster analyses and spatial models, among others. In addition, fast advances in technology suggest that the number of sequenced cells will dramatically increase in the near future (e.g. the one described in [19]), hence we foresee that a parallel implementation of the algorithm (e.g. using graphical processing units) might be necessary to cope with such large datasets more efficiently.

## Supporting Information

S1 Text Prior specification and posterior propriety. Description of the prior distribution employed for the analysis. As the suggested prior is *improper* (i.e. the integral over the parameter space is not finite), posterior propriety must be verified before performing inference. Nonetheless, we show that a sufficient condition for posterior existence is that each biological gene must be expressed (positive count) in at least one cell.
(PDF)

S2 Text Implementation. Description of the algorithm employed for the implementation of Bayesian inference. This is based on the Adaptive Metropolis (AM) within Gibbs Sampling (GS) algorithm presented in [13]. Posterior inference was implemented in C++ and R via the Rcpp library [14]. An R package has been prepared and is available at: https://github.com/catavallejos/BASiCS.
(PDF)

S3 Text Implementation specification when analysing the mouse ESC dataset. Hyper-parameter values and other input quantities required for the algorithm described in S2 Text. Includes Figures S1 and S2.
(PDF)

S4 Text EFDR and EFNR related to highly (and lowly) variable genes detection for a range of variance contribution thresholds. Optimal evidence thresholds (when the EFDR and the EFNR coincide) the associated values of EFDR (=EFNR) are provided. We also display the total number of genes that would be detected as HVG or LVG for each set of thresholds. Includes Table S1.
(PDF)

S5 Text Gene ontology enrichment analysis of highly (and lowly) variable genes among the analysed mouse ESC dataset. Results of gene ontology enrichment analysis among the genes detected as highly (and lowly) variable by BASiCS and the methods described in [16] and [5]. Includes Table S2.
(PDF)

S6 Text On the interpretation of the cell-specific mRNA content normalising constants. Using a cell-cycle annotated dataset we illustrate how the cell-specific mRNA content normalising constants *ϕ*_*j*_ can partially capture the cell cycle effect. Includes Figure S3.
(PDF)

S7 Text Computational cost. A summary of the computational cost of the MCMC algorithm using simulated datasets with a variety of numbers of cells and genes. Includes Figure S4.
(PDF)

S8 Text Analysis of Zeisel et al (2015) dataset. Illustrates the performance of BASiCS when analysing a larger scale dataset, including samples from several sub-populations. Includes Table S3 and Figures S5–S8.
(PDF)

S1 Data Analysed dataset and analysis code. (ZIP)
