# Supplementary material for: BASiCS: Bayesian Analysis of Single-Cell Sequencing Data
Source: PLoS Comput Biol. 2015 Jun 24;11(6):e1004333. doi: 10.1371/journal.pcbi.1004333 (PMC4480965; doi:10.1371/journal.pcbi.1004333)
Supplement: S1 Text — Description of the prior distribution employed for the analysis. As the suggested prior is improper (i.e. the integral over the parameter space is not finite), posterior propriety must be verified before performing inference. Nonetheless, we show that a sufficient condition for posterior existence is that each biological gene must be expressed (positive count) in at least one cell. (PDF) [file pcbi.1004333.s001.pdf]

# S1 Text: Prior specification and posterior propriety. BASiCS: Bayesian Analysis of Single-Cell Sequencing Data

Catalina A. Vallejos(1),(2), John C. Marioni(2), Sylvia Richardson(1)

(1) MRC Biostatistics Unit, Institute of Public Health, University Forvie Site, Robinson Way, Cambridge CB2 0SR, United Kingdom
(2) EMBL European Bioinformatics Institute, Cambridge, CB10 1SD, United Kingdom

We assume prior independence between all model parameters. In principle, a prior for the biological gene-specific normalised expression rates $\mu_1, \ldots, \mu_{q_0}$ could be elicited from an expert's opinion. Nonetheless, this is not trivial in this context, especially when $q_0$ is large and/or novel genes are being analysed. Hence, as an alternative, we adopt the improper *non-informative* prior:

$$\pi(\mu_1, \ldots, \mu_{q_0}) \propto \prod_{i=1}^{q_0} \mu_i^{-1}, \tag{S1}$$

which induces, over the real line, a uniform prior for each $\log(\mu_i)$ (this prior is improper because the integral of $\pi(\mu_1, \ldots, \mu_{q_0})$ over the support of $(\mu_1, \ldots, \mu_{q_0})$ is not finite, i.e. it is not a well defined density function). In the absence of reliable prior information, the Bayesian literature widely recommends this prior for Poisson rates [1]. We assume proper prior distributions for all other parameters with $\delta_1, \ldots, \delta_{q_0} \overset{\text{iid}}{\sim}$ Gamma$(a_\delta, b_\delta)$, $\kappa_2, \ldots, \kappa_n \overset{\text{iid}}{\sim}$ Normal$(0, \sigma^2_\kappa)$, $s_1, \ldots, s_n \overset{\text{iid}}{\sim}$ Gamma$(a_s, b_s)$ and $\theta \sim$ Gamma$(a_\theta, b_\theta)$ for fixed hyper-parameter values $a_\delta, b_\delta$, $s^2_\kappa, a_s, b_s, a_\theta$ and $b_\theta$. The analysis of simulated and real datasets suggested that the choice of these hyper-parameters does not have major consequences in posterior inference (this is illustrated in Fig S1. in S2 Text, using the mouse ESC dataset analysed throughout the paper). The proposed prior can be represented as

$$\pi(\mu_1, \ldots, \mu_{q_0}, \delta_1, \ldots, \delta_{q_0}, \theta) \propto \left[\prod_{i=1}^{q_0} \pi(\mu_i)\right] \left[\prod_{i=1}^{q_0} \pi(\delta_i)\right] \left[\prod_{j=2}^{n} \pi(\kappa_j)\right] \left[\prod_{j=1}^{n} \pi(s_j)\right] \pi(\theta), \tag{S2}$$

where $\pi(\mu_i) \propto \mu_i^{-1}$ and all the other components induce proper prior distributions for the corresponding parameters. While using *non informative* priors is a convenient solution that avoids the need of prior elicitation (the prior of $\mu_i$, which is the improper part, does not require the elicitation of hyper-parameters), it has the associated risk of producing invalid posterior inference. Hence, as the prior in (S2) is improper, posterior propriety must be verified. However, as shown by the following theorem, a sufficient condition for posterior existence is that each biological gene must be expressed (positive count) in at least one cell.

**Theorem 1**

Let $\{x_{ij} : i = 1, \ldots, q, j = 1, \ldots, n\}$ be $nq$ observations generated by the model in equations (2) and (3) of the main paper. Assume the prior in (S2). The joint posterior distribution of all model parameters is well defined if and only if $\min_{i \in \{1, \ldots, q_0\}} \left\{\sum_{j=1}^{n} x_{ij}\right\} > 0$, i.e. if and only if each biological gene is expressed in at least one cell.

*Proof.* Let $f_1(\cdot|s_j, \theta)$ and $f_2(\cdot|\delta_i)$ represent the densities associated to the random effects $\nu_j$ and $\rho_{ij}$, respectively (in BASiCS, we assume these densities belong to a Gamma family but this theorem is general, being also valid if other distributions are adopted for the random effects). The posterior distribution of all model parameters is well defined if and only if the marginal likelihood of the model (after integrating all model parameters

with respect to their prior) is finite. Here, it is given by

$$\int \left\{ \left[\prod_{i=1}^{q} L_i\right] \left[\prod_{j=1}^{n} f_1(\nu_j|s_j,\theta)\right] \left[\prod_{i=1}^{q_0}\prod_{j=1}^{n} f_2(\rho_{ij}|\delta_i)\right] \left[\prod_{i=1}^{q_0} \pi(\mu_i)\right] \left[\prod_{i=1}^{q_0} \pi(\delta_i)\right] \left[\prod_{j=2}^{n} \pi(\kappa_j)\right] \times \right.$$
$$\left. \left[\prod_{j=1}^{n} \pi(s_j)\right] \pi(\theta) \right\} \prod_{i=1}^{q_0} d\mu_i \prod_{i=1}^{q_0} d\delta_i \prod_{i=1}^{q_0}\prod_{j=1}^{n} d\rho_{ij} \prod_{j=1}^{n} d\nu_j \prod_{j=1}^{n} d\phi_j \prod_{j=1}^{n} ds_j \, d\theta, \tag{S3}$$

with

$$L_i = \prod_{j=1}^{n} \mathrm{P}(X_{ij} = x_{ij}|\mu_i, \phi_j, \nu_j, \rho_{ij}) = \begin{cases} \displaystyle\prod_{j=1}^{n} \frac{(\phi_j \nu_j \mu_i \rho_{ij})^{x_{ij}} \, e^{-\phi_j \nu_j \mu_i \rho_{ij}}}{x_{ij}!}, & i = 1, \ldots, q_0; \\ \displaystyle\prod_{j=1}^{n} \frac{(\nu_j \mu_i)^{x_{ij}} \, e^{-\nu_j \mu_i}}{x_{ij}!}, & i = q_0 + 1, \ldots, q, \end{cases} \tag{S4}$$

and $\phi_j$'s defined as a function of $\kappa_j$'s as in equation (11) of the main paper. Using Fubini's theorem in (S3) and integrating first with respect to the $\mu_1, \ldots, \mu_{q_0}$, (S3) is equal to

$$\int \left\{ \left[\prod_{i=q_0+1}^{q} L_i\right] \left[\prod_{i=1}^{q_0}\prod_{j=1}^{n} L^*_{ij}\right] \left[\prod_{j=1}^{n} f_1(\nu_j|s_j,\theta)\right] \left[\prod_{i=1}^{q_0}\prod_{j=1}^{n} f_2(\rho_{ij}|\delta_i)\right] \left[\prod_{i=1}^{q_0} \pi(\delta_i)\right] \times \right.$$
$$\left. \left[\prod_{j=2}^{n} \pi(\kappa_j)\right] \left[\prod_{j=1}^{n} \pi(s_j)\right] \pi(\theta) \right\} \prod_{i=1}^{q_0} d\delta_i \prod_{i=1}^{q_0}\prod_{j=1}^{n} d\rho_{ij} \prod_{j=1}^{n} d\nu_j \prod_{j=1}^{n} d\phi_j \prod_{j=1}^{n} ds_j \, d\theta, \tag{S5}$$

with

$$L^*_{ij} = \prod_{i=1}^{q_0}\prod_{j=1}^{n} \left[\frac{(\phi_j \nu_j \rho_{ij})^{x_{ij}}}{x_{ij}!}\right] \int_0^\infty \mu_i^{\sum_{j=1}^n x_{ij} - 1} \, e^{-\mu_i \sum_{j=1}^n \phi_j \nu_j \rho_{ij}} \, d\mu_i \tag{S6}$$

$$= \frac{\Gamma\left(\displaystyle\sum_{j=1}^{n} x_{ij}\right) \displaystyle\prod_{i=1}^{q_0}\prod_{j=1}^{n} (\phi_j \nu_j \rho_{ij})^{x_{ij}}}{\displaystyle\prod_{i=1}^{q_0}\prod_{j=1}^{n} x_{ij}! \left(\displaystyle\sum_{j=1}^{n} \phi_j \nu_j \rho_{ij}\right)^{\sum_{j=1}^n x_{ij}}}, \quad \text{provided that } \textstyle\sum_{j=1}^n x_{ij} > 0. \tag{S7}$$

Therefore, (S3) is not finite unless $\min_{i \in \{1,\ldots,q_0\}} \left\{\sum_{j=1}^n x_{ij}\right\} > 0$. In addition, as each $L_i$ is a product of Poisson probabilities, $\prod_{i=q_0+1}^{q} L_i \leq 1$. Hence, replacing (S7) in (S5), (S3) has an upper bound proportional to

$$\int \left\{ \left[\prod_{i=1}^{q_0}\prod_{j=1}^{n} \left(\frac{\phi_j \nu_j \rho_{ij}}{A_i}\right)^{x_{ij}}\right] \left[\prod_{j=1}^{n} f_1(\nu_j|s_j,\theta)\right] \left[\prod_{i=1}^{q_0}\prod_{j=1}^{n} f_2(\rho_{ij}|\delta_i)\right] \left[\prod_{i=1}^{q_0} \pi(\delta_i)\right] \times \right.$$
$$\left. \left[\prod_{j=2}^{n} \pi(\kappa_j)\right] \left[\prod_{j=1}^{n} \pi(s_j)\right] \pi(\theta) \right\} \prod_{i=1}^{q_0} d\delta_i \prod_{i=1}^{q_0}\prod_{j=1}^{n} d\rho_{ij} \prod_{j=1}^{n} d\nu_j \prod_{j=1}^{n} d\phi_j \prod_{j=1}^{n} ds_j \, d\theta, \tag{S8}$$

with $A_i = \sum_{j=1}^n \phi_j \nu_j \rho_{ij}$. As $\phi_j \nu_j \rho_{ij}/A_i \leq 1$ for all $i = 1, \ldots, q_0$ and $j = 1, \ldots, n$, (S8) is bounded above by

$$\left[\prod_{j=1}^{n} \int_0^\infty f_1(\nu_j|s_j,\theta) \, d\nu_j\right] \left[\prod_{i=1}^{q_0}\prod_{j=1}^{n} \int_0^\infty f_2(\rho_{ij}|\delta_i) \, d\rho_{ij}\right] \left[\prod_{i=1}^{q_0} \int_0^\infty \pi(\delta_i) \, d\delta_i\right] \times$$
$$\left[\prod_{j=2}^{n} \int_{-\infty}^\infty \pi(\kappa_j) \, d\kappa_j\right] \left[\prod_{j=1}^{n} \int_0^\infty \pi(s_j) \, ds_j\right] \left[\int_0^\infty \pi(\theta) \, d\theta\right]. \tag{S9}$$

Hence, because $\pi(\delta_i)$, $\pi(\kappa_j)$, $\pi(s_j)$ and $\pi(\theta)$ define proper prior densities, (S9) is equal to 1 and, consequently, (S3) is finite. □

## References

[1] Jeffreys H (1967) Theory of Probability. Clarendon Press, third edition.
