# Supplementary material for: BASiCS: Bayesian Analysis of Single-Cell Sequencing Data
Source: PLoS Comput Biol. 2015 Jun 24;11(6):e1004333. doi: 10.1371/journal.pcbi.1004333 (PMC4480965; doi:10.1371/journal.pcbi.1004333)
Supplement: S2 Text — Description of the algorithm employed for the implementation of Bayesian inference. This is based on the Adaptive Metropolis (AM) within Gibbs Sampling (GS) algorithm presented in [13]. Posterior inference was implemented in C++ and R via the Rcpp library [14]. An R package has been prepared and is available at: https://github.com/catavallejos/BASiCS. (PDF) [file pcbi.1004333.s002.pdf]

S2 Text: Implementation
BASiCS: Bayesian Analysis of Single-Cell Sequencing Data

Catalina A. Vallejos$^{(1),(2)}$, John C. Marioni$^{(2)}$, Sylvia Richardson$^{(1)}$

(1) MRC Biostatistics Unit, Institute of Public Health, University Forvie Site, Robinson Way, Cambridge CB2 0SR, United Kingdom
(2) EMBL European Bioinformatics Institute, Cambridge, CB10 1SD, United Kingdom

Bayesian inference is implemented using an Adaptive Metropolis (AM) within Gibbs Sampling (GS) algorithm as introduced by [1]. If all model parameters and random effects are sampled throughout the algorithm, most of the full conditionals are conjugate, with AM updates only being required for $\delta_1, \ldots, \delta_{q_0}$ and θ. Although this can be easily implemented, the algorithm is affected by a very poor mixing, mainly related to the updates of the ρ_{ij}'s (particularly critical when n and/or q_0 are large). To overcome this problem, we implemented Bayesian inference based on the marginal model obtained after integrating out the ρ_{ij}'s, i.e.

$$X_{ij}|\mu_i, \delta_i, \phi_j, \nu_j, \theta \sim \begin{cases} \text{Neg-Binomial}\left(\delta_i^{-1}, \frac{\phi_j \nu_j \mu_i}{\phi_j \nu_j \mu_i + \delta_i^{-1}}\right), & i = 1, \ldots, q_0, j = 1, \ldots, n; \\ \text{Poisson}(\nu_j \mu_i), & i = q_0 + 1, \ldots, q, j = 1, \ldots, n, \end{cases} \tag{S1}$$

for which the associated likelihood function is given by

$$\left[\prod_{i=1}^{q_0}\prod_{j=1}^{n} \frac{\Gamma(x_{ij} + 1/\delta_i)}{\Gamma(1/\delta_i) x_{ij}!} \left(\frac{1/\delta_i}{\phi_j \nu_j \mu_i + 1/\delta_i}\right)^{1/\delta_i} \left(\frac{\phi_j \nu_j \mu_i}{\phi_j \nu_j \mu_i + 1/\delta_i}\right)^{x_{ij}}\right] \left[\prod_{i=q_0+1}^{q}\prod_{j=1}^{n} \frac{(\nu_j \mu_i)^{x_{ij}}}{x_{ij}!} e^{-\nu_j \mu_i}\right]. \tag{S2}$$

By doing this, the mixing of the sampler was substantially improved. However, the conjugacy of the full conditionals required for the GS is lost. In fact, the full conditionals required for the GS implementation are given by

$$\pi(\mu_i|\cdots) \propto \frac{\mu_i^{\sum_{j=1}^n x_{ij} - 1}}{\prod_{j=1}^n (\phi_j \nu_j \mu_i + 1/\delta_i)^{x_{ij} + 1/\delta_i}}, \qquad i = 1, \ldots, q_0, \tag{S3}$$

$$\pi(\nu_j|\cdots) \propto \left[\prod_{i=1}^{q_0} \frac{\nu_j^{x_{ij}}}{(\phi_j \nu_j \mu_i + 1/\delta_i)^{x_{ij}+1/\delta_i}}\right] \left[\prod_{i=q_0+1}^{q} \nu_j^{x_{ij}} e^{-\nu_j \mu_i}\right] \nu_j^{(1/\theta)-1} e^{-\nu_j/(\theta s_j)}, \tag{S4}$$

$$= \frac{\nu_j^{\sum_{i=1}^q x_{ij} + 1/\theta - 1}}{\prod_{i=1}^{q_0} (\phi_j \nu_j \mu_i + 1/\delta_i)^{x_{ij}+1/\delta_i}} e^{-\nu_j \left(\sum_{i=q_0+1}^q \mu_i + 1/(\theta s_j)\right)}, \qquad j = 1, \ldots, n, \tag{S5}$$

$$\pi(\theta|\cdots) \propto \frac{\left(\prod_{j=1}^n (\nu_j/s_j)\right)^{1/\theta}}{\Gamma^n(1/\theta)} \theta^{a_\theta - (n/\theta) - 1} e^{-(1/\theta)\sum_{j=1}^n (\nu_j/s_j) - b_\theta \theta} \tag{S6}$$

$$\pi(\delta_i|\cdots) \propto \left[\prod_{j=1}^n \frac{\Gamma(x_{ij} + 1/\delta_i)}{\Gamma(1/\delta_i)} \frac{(1/\delta_i)^{1/\delta_i}}{(\phi_j \nu_j \mu_i + 1/\delta_i)^{x_{ij}+1/\delta_i}}\right] \delta_i^{a_\delta - 1} e^{-b_\delta \delta_i}, \tag{S7}$$

$$= \frac{1}{\Gamma^n(1/\delta_i)} \left[\prod_{j=1}^n \frac{\Gamma(x_{ij} + 1/\delta_i)}{(\phi_j \nu_j \mu_i + 1/\delta_i)^{x_{ij}+1/\delta_i}}\right] \delta_i^{a_\delta - (n/\delta_i) - 1} e^{-b_\delta \delta_i}, \quad i = 1, \ldots, q_0, \tag{S8}$$

$$\pi(\kappa_j|\cdots) \propto \frac{\phi_j^{\sum_{i=1}^{q_0} x_{ij}}}{\prod_{i=1}^{q_0} (\phi_j \nu_j \mu_i + 1/\delta_i)^{x_{ij}+1/\delta_i}} e^{-\kappa_j^2/(2\sigma_\kappa^2)}, \tag{S9}$$

ϕ_j as in eq. (11) of the paper $j = 2, \ldots, n$

$$\pi(s_j|\cdots) \propto s_j^{a_s - (1/\theta) - 1} \exp\left\{-\frac{\nu_j}{s_j \theta} - s_j b_s\right\}, \qquad j = 1, \ldots, n \tag{S10}$$

Although the expression in (S10) is proportional to a Generalized Inverse Gaussian (GIG) density with parameters $a_s - 1/\theta$, $2b_s$ and $2\nu_j/\theta$ (the GIG distribution is parametrised as in [2], p. 478)[1], AM updates are implemented when sampling all other model parameters. We implemented the adaptive proposals, such that the adaptation stops after a fixed number of iterations (to be specified by the user). Because of numerical issues related to the evaluation of the Gamma function in extremely large values, we truncated the value of θ to be larger than 1×10^{-4}. Nonetheless, this does not have practical consequences in posterior inference, because the range $(0, 1 \times 10^{-4})$ does not discard interesting cases.

Bayesian inference was implemented using a combination of `R` and C++ via the library `Rcpp`. Code is provided as supplementary material and available at `https://github.com/catavallejos/BASiCS`.

References

[1] Roberts GO, Rosenthal JS (2009) Examples of adaptive MCMC. Journal of Computational and Graphical Statistics 18: 349-367.

[2] Devroye L (1986) Non-Uniform Random Variable Generation. Springer.

[1]Draws from this distribution are generated using the C code provided by Ester Pantaleo and Robert B. Gramacy (https://r-forge.r-project.org/scm/viewvc.php/pkg/ghyp/src/rgig.c?view=markup&root=rmetrics&pathrev=4804).
