# Supplementary material for: BASiCS: Bayesian Analysis of Single-Cell Sequencing Data
Source: PLoS Comput Biol. 2015 Jun 24;11(6):e1004333. doi: 10.1371/journal.pcbi.1004333 (PMC4480965; doi:10.1371/journal.pcbi.1004333)
Supplement: S3 Text — Hyper-parameter values and other input quantities required for the algorithm described in S2 Text. Includes Figures S1 and S2. (PDF) [file pcbi.1004333.s003.pdf]

S3 Text: Implementation specification when analysing mouse ESC dataset BASiCS: Bayesian Analysis of Single-Cell Sequencing Data

Catalina A. Vallejos[(1),(2)], John C. Marioni[(2)], Sylvia Richardson[(1)]

(1) MRC Biostatistics Unit, Institute of Public Health, University Forvie Site, Robinson Way, Cambridge CB2 0SR, United Kingdom
(2) EMBL European Bioinformatics Institute, Cambridge, CB10 1SD, United Kingdom

We conducted the analysis using different configurations of hyper-parameter values, with a range where the prior expectations of θ and δ_i's vary from 0.4 to 2.5 and the corresponding prior variances vary from 0.4 to 6.25. For each set of hyper-parameters values, we ran $N = 20,000$ iterations of the MCMC algorithm, storing draws every 10 iterations and ignoring an initial burn-in period of 10,000 iterations (hence, results are shown in terms of 1,000 iterations). Changes in hyper-parameter values (within the employed range) produced negligible differences in posterior inference (see Fig S1.). Therefore, we show numerical results on the basis of a single configuration of hyper-parameter values (all equal to 1). Trace-plots and auto-correlation plots (some of which are shown in Fig S2.) suggest a good mixing of the chains. Regarding the adaptive proposals, we stopped the adaptation after 10,000 iterations, hence the results presented here are based on constant proposal variances.

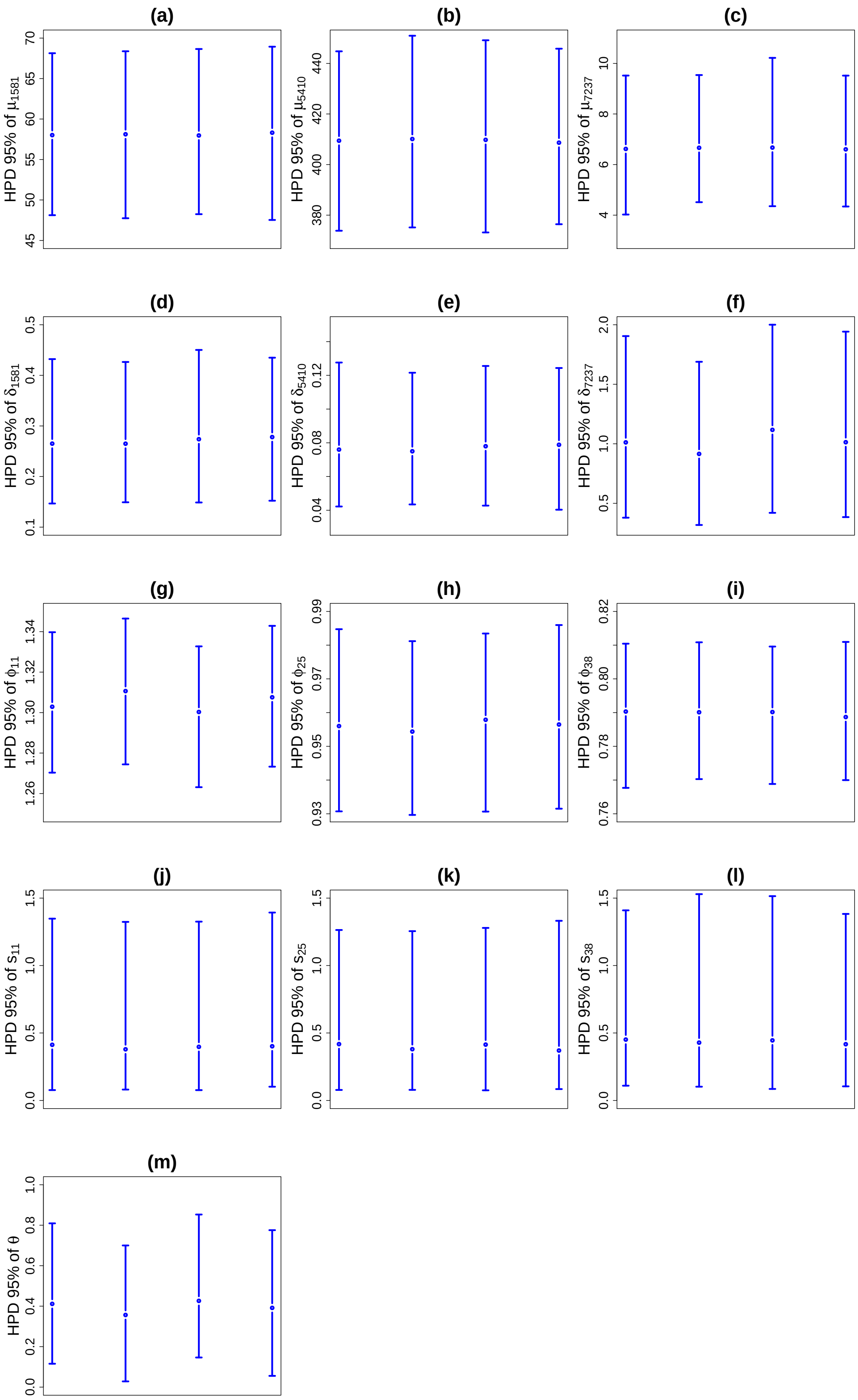

Figure S1: **Sensitivity to prior hyper parameters.** For the mouse ES dataset analysed on the main paper. Vertical lines represent the 95% high posterior density interval (dot located at the posterior median) for 4 different combinations of hyper parameter values (within the range described in this supplementary text). First and second rows: μ_i and δ_i for 3 randomly selected genes, respectively. Third and fourth rows: ϕ_j and s_j for 3 randomly selected cells, respectively. Fifth row: θ. All these hyper parameters configurations produced virtually the same posterior inference for all model parameters.

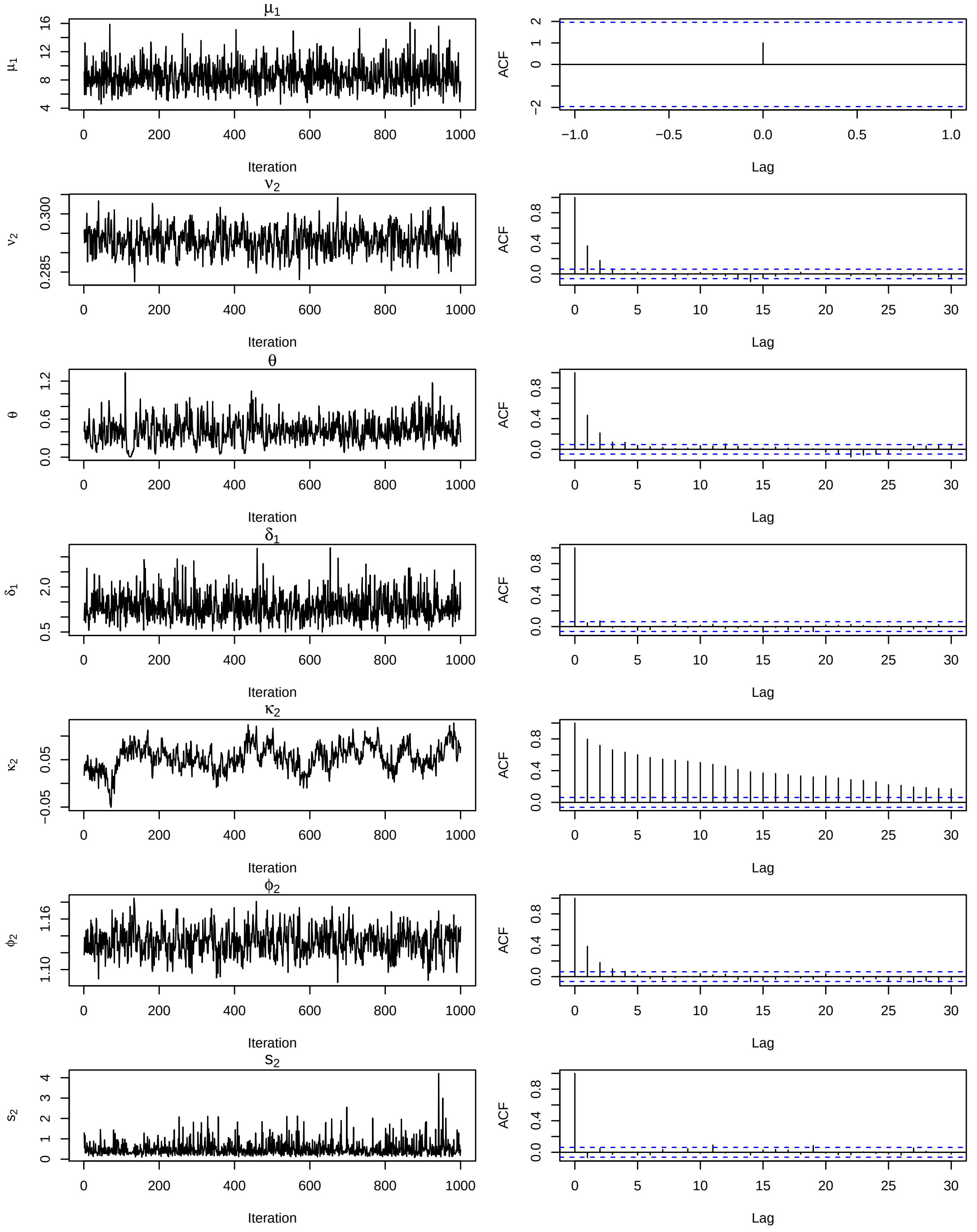

Figure S2: For the mouse ES dataset analysed on the main paper. Trace-plots (left) and auto-correlation plots (right) for μ_1, ν_2, θ, δ_1, κ_2, ϕ_2 and s_2. Trace-plots suggest convergence of the chains. The chains of the κ_j's mix less well, however mixing is substantially improved when presenting the results in terms of the ϕ_j's.
