# Supplementary material for: BASiCS: Bayesian Analysis of Single-Cell Sequencing Data
Source: PLoS Comput Biol. 2015 Jun 24;11(6):e1004333. doi: 10.1371/journal.pcbi.1004333 (PMC4480965; doi:10.1371/journal.pcbi.1004333)
Supplement: S4 Text — Optimal evidence thresholds (when the EFDR and the EFNR coincide) the associated values of EFDR (=EFNR) are provided. We also display the total number of genes that would be detected as HVG or LVG for each set of thresholds. Includes Table S1. (PDF) [file pcbi.1004333.s004.pdf]

S4 Text: EFDR and EFNR related to highly (and lowly) variable genes detection for a range of variance contribution thresholds.
BASiCS: Bayesian Analysis of Single-Cell Sequencing Data

Catalina A. Vallejos(1),(2), John C. Marioni(2), Sylvia Richardson(1)

(1) MRC Biostatistics Unit, Institute of Public Health, University Forvie Site, Robinson Way, Cambridge CB2 0SR, United Kingdom
(2) EMBL European Bioinformatics Institute, Cambridge, CB10 1SD, United Kingdom

Table S1: Optimal evidence thresholds (when the EFDR and the EFNR coincide) the associated values of EFDR (=EFNR) are provided. We also display the total number of genes that would be detected as HVG or LVG for each set of thresholds.

Detecting	Var. Threshold	Optimal EFDR (=EFNR)	Optimal evidence threshold	Detected genes
HVG	70%	17.300%	0.6300	1726
	71%	17.071%	0.6325	1459
	72%	16.597%	0.6575	1199
	73%	16.048%	0.6750	961
	74%	15.496%	0.7000	756
	75%	14.601%	0.7175	565
	76%	13.767%	0.7375	416
	77%	12.592%	0.7600	288
	78%	11.389%	0.7725	196
	**79%**	**10.080%**	**0.7925**	**133**
	80%	8.878%	0.8200	86
LVG	35%	7.854%	0.8325	271
	36%	8.176%	0.8200	308
	37%	8.585%	0.8150	354
	38%	8.891%	0.8125	403
	39%	9.334%	0.8000	465
	40%	9.589%	0.7850	523
	**41%**	**9.965%**	**0.7650**	**589**
	42%	10.391%	0.7525	660
	43%	10.759%	0.7375	732
	44%	11.220%	0.7275	816
	45%	11.517%	0.7200	899
