# Supplementary material for: BASiCS: Bayesian Analysis of Single-Cell Sequencing Data
Source: PLoS Comput Biol. 2015 Jun 24;11(6):e1004333. doi: 10.1371/journal.pcbi.1004333 (PMC4480965; doi:10.1371/journal.pcbi.1004333)
Supplement: S5 Text — Results of gene ontology enrichment analysis among the genes detected as highly (and lowly) variable by BASiCS and the methods described in [16] and [5]. Includes Table S2. (PDF) [file pcbi.1004333.s005.pdf]

S5 Text: Gene ontology enrichment analysis of highly (and lowly) variable genes among the analysed mouse ESC dataset.
BASiCS: Bayesian Analysis of Single-Cell Sequencing Data

Catalina A. Vallejos(1),(2), John C. Marioni(2), Sylvia Richardson(1)

(1) MRC Biostatistics Unit, Institute of Public Health, University Forvie Site, Robinson Way, Cambridge CB2 0SR, United Kingdom
(2) EMBL European Bioinformatics Institute, Cambridge, CB10 1SD, United Kingdom

Table S2: Gene ontology (GO) enrichment analysis of genes detected as highly (or lowly) variably by BASiCS and the methods described in [1] and [2]. Only the 5 GO categories with smallest p-values are displayed in each case.

**Method**	**GO term**	**Annotated**	**Significant**	**Expected**	p**-value**
BASiCS (LVG)	Translation	340	101	25.87	1.8×10^{-19}
	ATP synthesis coupled proton transport	14	11	1.07	1.3×10^{-10}
	Translational initiation	68	26	5.17	4.9×10^{-10}
	Binding of sperm to zona pellucida	10	8	0.76	4.2×10^{-8}
	Translational elongation	33	13	2.51	3.4×10^{-7}
BASiCS (HVG)	Cell differentiation	1017	25	12.43	1.2×10^{-3}
	Regulation of epidermis development	19	3	0.23	1.5×10^{-3}
	Brown fat cell differentiation	8	2	0.1	3.9×10^{-3}
	Pyrimidine nucleobase metabolic process	8	2	0.1	3.9×10^{-3}
	Pyrimidine-containing compound catabolic process	8	2	0.1	3.9×10^{-3}
Islam (HVG)	Negative regulation of cell proliferation	204	16	3.15	1.3×10^{-6}
	Nitric oxide mediated signal transduction	5	3	0.08	3.5×10^{-5}
	Cellular response to metal ion	21	4	0.32	2.6×10^{-4}
	Positive regulation of blood pressure	9	3	0.14	2.8×10^{-4}
	Regulation of cell growth	114	8	1.76	3.5×10^{-4}
Brennecke (HVG)	Lung development	75	25	10.04	7.1×10^{-6}
	Anterior/posterior axis specification	26	12	3.48	4.9×10^{-5}
	Somitogenesis	24	11	3.21	1.1×10^{-4}
	Regulation of cell migration	165	47	22.08	1.5×10^{-4}
	Regulation of embryonic development	33	13	4.42	1.7×10^{-4}

References

[1] Islam S, Zeisel A, Joost S, La Manno G, Zajac P, et al. (2014) Quantitative single-cell RNA-seq with unique molecular identifiers. Nature Methods 11: 163-166.

[2] Brennecke P, Anders S, Kim JK, Kołodziejczyk AA, Zhang X, et al. (2013) Accounting for technical noise in single-cell RNA-seq experiments. Nature Methods 10: 1093-1095.
