# Supplementary material for: BASiCS: Bayesian Analysis of Single-Cell Sequencing Data
Source: PLoS Comput Biol. 2015 Jun 24;11(6):e1004333. doi: 10.1371/journal.pcbi.1004333 (PMC4480965; doi:10.1371/journal.pcbi.1004333)
Supplement: S6 Text — Using a cell-cycle annotated dataset we illustrate how the cell-specific mRNA content normalising constants ϕ j can partially capture the cell cycle effect. Includes Figure S3. (PDF) [file pcbi.1004333.s006.pdf]

S6 Text: On the interpretation of the cell-specific mRNA content normalising constants.
BASiCS: Bayesian Analysis of Single-Cell Sequencing Data

Catalina A. Vallejos[(1),(2)], John C. Marioni[(2)], Sylvia Richardson[(1)]

(1) MRC Biostatistics Unit, Institute of Public Health, University Forvie Site, Robinson Way, Cambridge CB2 0SR, United Kingdom
(2) EMBL European Bioinformatics Institute, Cambridge, CB10 1SD, United Kingdom

It is common to observe a large range of cell sizes (here we use "cell size" as a synonym for total mRNA content) within populations of cells. Part of this heterogeneity is explained by the different stages of the cell cycle at which the cells have been captured (cells tend to become larger in later stages of the cell cycle). The cell-specific mRNA content normalising constants ϕ_j partially capture the cell cycle effect. As a validation of this idea, we analysed the mouse ESC dataset described in [1], where the cell cycle stage of the analysed cells has been recorded. For these data, BASiCS suggests a strong separation of the cells captured during phases G1 or S from those cells captured while in G2 or M phases, the latter being larger cells (see Figure S3 of this supplementary material).

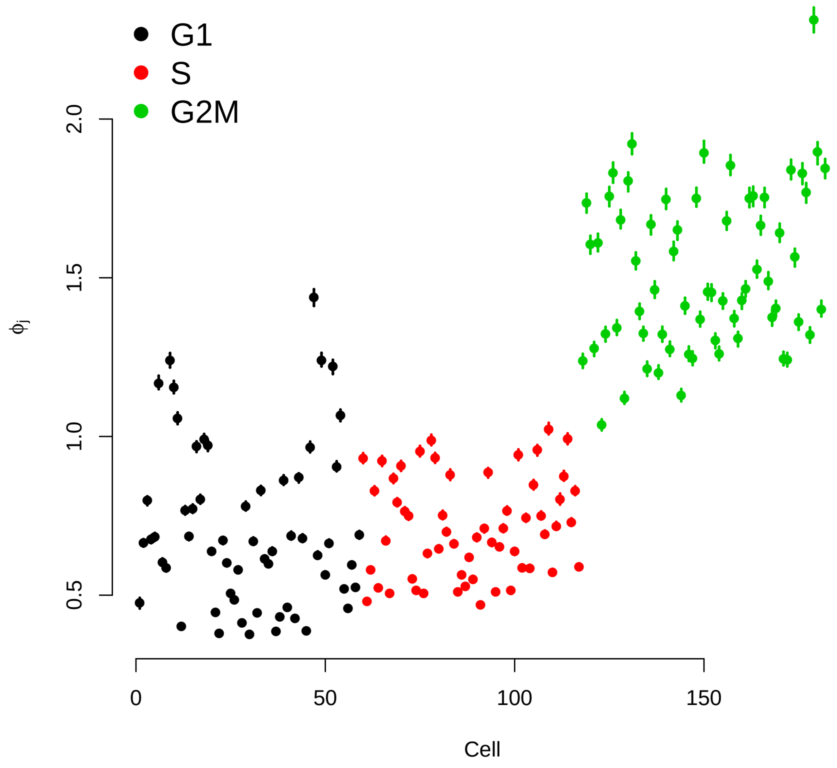

Figure S3: Posterior estimates of cell-to-cell mRNA content normalising constants for the mouse ESC dataset described in [1]. BASiCS suggests a strong separation of the cells being in phases G1 or S with respect to those cells being in G2 or M phases, the latter being larger cells.

References

[1] Buettner F, Natarajan KN, Casale FP, Proserpio V, Scialdone A, et al. (2015) Computational analysis of cell-to-cell heterogeneity in single-cell rna-sequencing data reveals hidden subpopulations of cells. Nature biotechnology .
