# Supplementary material for: BASiCS: Bayesian Analysis of Single-Cell Sequencing Data
Source: PLoS Comput Biol. 2015 Jun 24;11(6):e1004333. doi: 10.1371/journal.pcbi.1004333 (PMC4480965; doi:10.1371/journal.pcbi.1004333)
Supplement: S7 Text — A summary of the computational cost of the MCMC algorithm using simulated datasets with a variety of numbers of cells and genes. Includes Figure S4. (PDF) [file pcbi.1004333.s007.pdf]

S7 Text: Computational cost.
BASiCS: Bayesian Analysis of Single-Cell Sequencing Data

Catalina A. Vallejos[(1),(2)], John C. Marioni[(2)], Sylvia Richardson[(1)]

(1) MRC Biostatistics Unit, Institute of Public Health, University Forvie Site, Robinson Way, Cambridge CB2 0SR, United Kingdom
(2) EMBL European Bioinformatics Institute, Cambridge, CB10 1SD, United Kingdom

The main computational cost of our method relates to the MCMC algorithm. Once the MCMC chains have been produced, downstream analyses such as the detection of highly and lowly variable genes require simple (and fast) post-processing of the output provided by the MCMC algorithm. To assess the computational cost of our method, we performed the analysis of simulated datasets generated from the model described in the manuscript using different numbers of cells n and genes q. For each combination of n and q, we fixed the number of spike-in genes equal to 50. For each dataset, we ran the MCMC algorithm for $N = 10,000$ iterations. For each combination of n and q, average results across 5 repetitions are displayed in Fig S4.. It can be seen that the computational complexity of our MCMC algorithm is approximately linear with respect to the number of cells as well as to the number of genes.

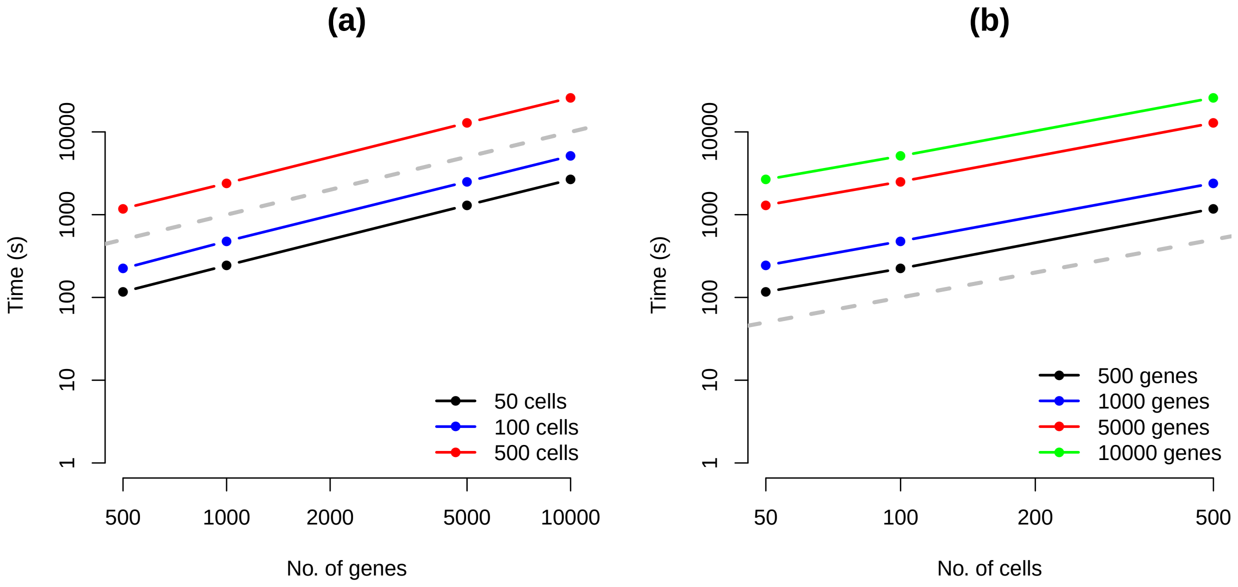

Figure S4: Computational cost. Running times (in seconds) required to generate $N = 10,000$ MCMC samples for different numbers of cells and genes. Running times correspond to average results across 5 repetitions and dashed grey lines located at “x=y”. It can be seen that the computational complexity of our MCMC algorithm is approximately linear with respect to the number of cells as well as to the number of genes.
