# Supplementary material for: BASiCS: Bayesian Analysis of Single-Cell Sequencing Data
Source: PLoS Comput Biol. 2015 Jun 24;11(6):e1004333. doi: 10.1371/journal.pcbi.1004333 (PMC4480965; doi:10.1371/journal.pcbi.1004333)
Supplement: S8 Text — Illustrates the performance of BASiCS when analysing a larger scale dataset, including samples from several sub-populations. Includes Table S3 and Figures S5–S8. (PDF) [file pcbi.1004333.s008.pdf]

S8 Text: Analysis of Zeisel et al (2015) dataset.
BASiCS: Bayesian Analysis of Single-Cell Sequencing Data

Catalina A. Vallejos(1),(2), John C. Marioni(2), Sylvia Richardson(1)

(1) MRC Biostatistics Unit, Institute of Public Health, University Forvie Site, Robinson Way, Cambridge CB2 0SR, United Kingdom
(2) EMBL European Bioinformatics Institute, Cambridge, CB10 1SD, United Kingdom

To illustrate the performance of BASiCS in a large scale dataset, we analysed the dataset described in [1], containing 3,005 cells from the mouse somatosensory cortex and hippocampal CA1 region. This is a UMI-based dataset. We excluded those genes with 300 or less counts across all cells (i.e. on average, less than 0.1 counts per cell), leaving only 11,814 genes (11,772 biological and 42 spike-in genes) for the analysis. We ran $N = 30,000$ iterations of the MCMC algorithm, storing draws every 15 iterations and ignoring an initial burn-in period of 15,000 iterations (hence, results are shown in terms of 1,000 iterations). We fix all hyper-parameters were equal to 1 (as discussed in S3 Text, this choice is not critical for the analysis). The current implementation of BASiCS is not designed to cope efficiently with such large datasets and therefore this MCMC run took around 6 days to be completed (we are currently working in a parallel programming version of BASiCS where this running time will be substantially reduced). However, most of the parameters' chains appear to converge after a short run (bellow 10,000 iterations) and it was only the poor mixing of the chain related to ϕ_1 (cell-specific mRNA content normalising constants for the first cell, which is left as a reference in order to fulfil the identifiability restriction $\sum_{j=1}^{n} \phi_j = n$) made necessary to increase the number of iterations. For shorter runs, we can still obtain stable results for all other model parameters in less than a day. Other downstream analyses such as the detection of highly and lowly variable genes were also stable for these shorter runs (our variance decomposition only depends on the median of $\phi_j s_j$ across all cells and therefore the value of ϕ_1 itself does not have a huge impact on the results).

The heterogeneity of the cells is reflected in the cell-specific mRNA content normalising constants ϕ_j. In fact, Fig S6(a). suggests a large variety of cell sizes and the range of the posterior estimates of ϕ_j is substantially larger than in the case of the mouse ESC dataset that was analysed in the manuscript. This is consistent with the evidence shown in [1], were the analysis indicated the existence of multiple distinct sub-populations of cells. In addition, Fig S6(b). indicates that the scale of the technical counts remains relatively stable among cells, which is expected when analysis UMI-based expression counts (because sequencing depth and other amplification biases are removed). However, the posterior distribution of θ (posterior median = 0.7, 95% HPD interval = (0.65, 0.75) suggests that the use of UMIs has not completely removed the effect of unexplained technical variation.

To detect highly variable genes, we explore three different values for the variance contribution threshold γ_H = 85%, 90% and 95%. A summary of these results is displayed in Table S3 and Figure S7. Unlike the mouse ESC dataset analysed in the manuscript, where highly variable genes were associated to low and medium expression rates, BASiCS does now detect highly variable genes across the whole range of expression. The difference in the results is mainly related to the nature of the samples. In fact, the mouse ESC dataset contained samples from a fairly homogeneous population of cells (instead of the heterogeneous mixture represented by the cells in the dataset presented by [1]) and highly expressed genes had stable expression across the cells because they related to core processes shared by all cells (acting as *housekeeping genes*).

There is a good agreement between our list of highly variable genes and the list of sub-population markers identified by [1] (see Fig S7(b).). In fact, for γ_H = 80%, *Acta2* (99%), *Aif1* (95%), *Aldoc* (96%), *Cldn5* (98%), *Gad1* (97%), *Mbp* (93%) and *Spink8* (87%) belong to both groups (posterior medians of σ_i, the percentage of variance related to a biological cell-to-cell heterogeneity component, are shown in parenthesis). The two remaining sub-population markers highlighted by [1], *Tbr1* (76%) and *Thy1* (64%), are not classified as highly variable genes by our method. To assess these results, we define *denoised* gene-cell-specific expression rates for which the effect of cell-specific normalisation and unexplained technical variation is removed. These are

given by

$$\Lambda_{ij} = \mu_i \rho_{ij}, \tag{S1}$$

for each gene i among the 11,772 biological genes. These quantities can be easily estimated based on a posterior sample of our model parameters, replacing ρ_{ij} by its posterior expectation and μ_i by its posterior median. As shown Fig S8., most of the variability in expression counts that has been observed for *Thy1* disappears after removing the effect to cell-specific normalisation and unexplained technical variation, illustrating that ignoring these components might mask the underlying composition of the cells.

Table S3: Highly variable genes detection for Zeisel et al (2015) dataset.

Var. Threshold	Optimal EFDR (=EFNR)	Optimal evidence threshold	No. of HVG
85%	0.74%	0.83	763
90%	0.62%	0.89	477
95%	0.36%	0.90	184

References

[1] Zeisel A, Muñoz-Manchado AB, Codeluppi S, Lönnerberg P, La Manno G, et al. (2015) Cell types in the mouse cortex and hippocampus revealed by single-cell rna-seq. Science 347: 1138–1142.

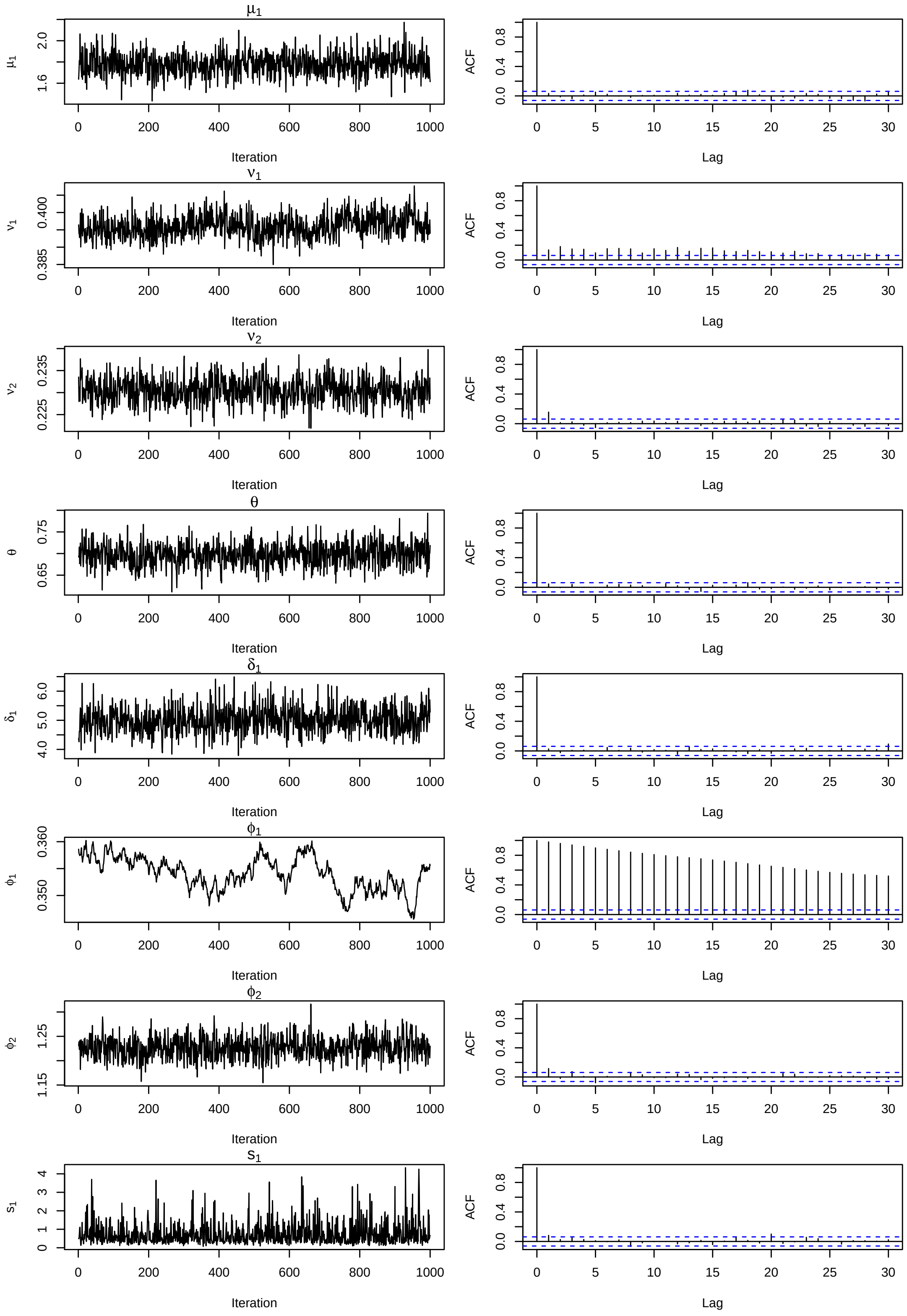

Figure S5: For the Zeisel et al (2015) dataset. Trace-plots (left) and auto-correlation plots (right) for μ_1, ν_1, ν_2, θ, δ_1, ϕ_1, ϕ_2 and s_1. Trace-plots suggest convergence of the chains. The chains related to ϕ_1 and ν_1 mix less well (with highly auto-correlated draws) yet the draws remain within a stable small range.

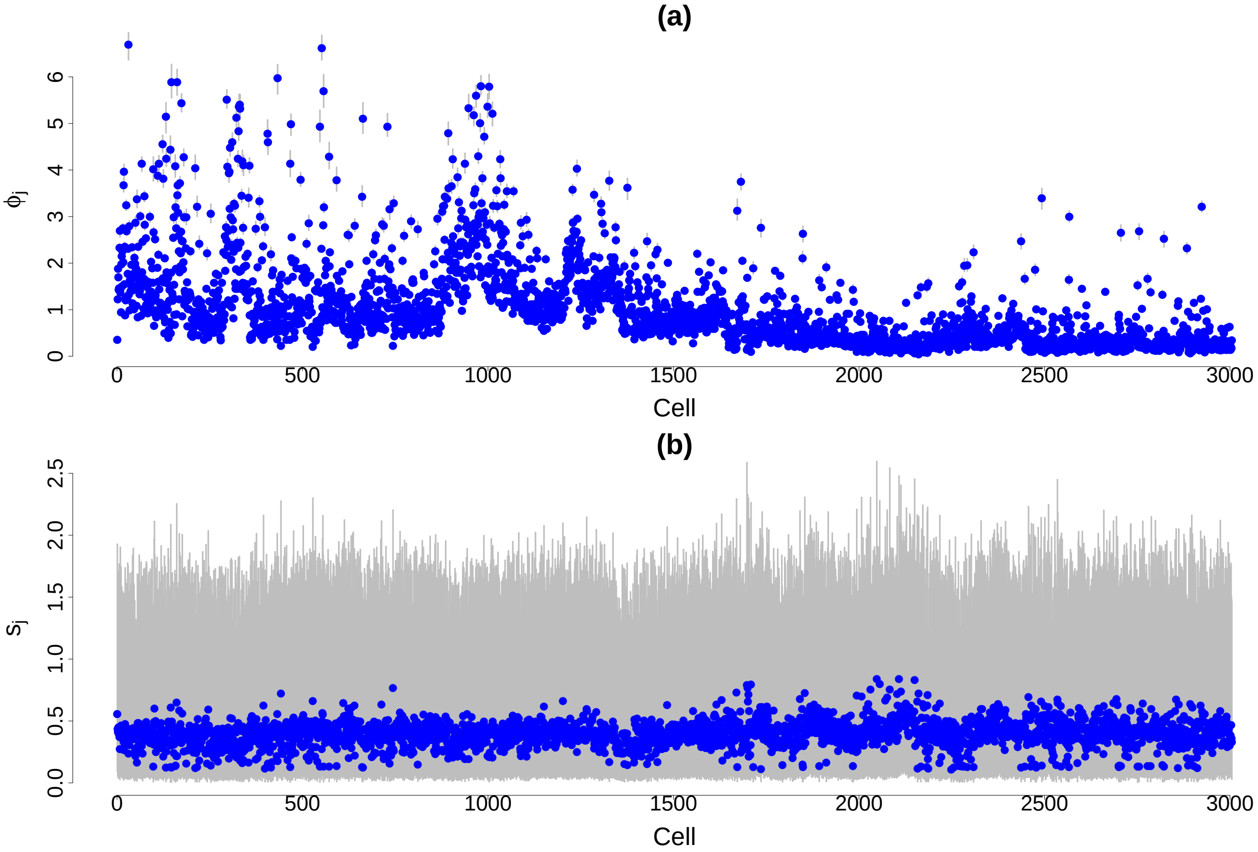

Figure S6: **Normalisation (Zeisel et al dataset).** (a) and (b): for each of the 3,005 cells, vertical lines represent the 95% high posterior density interval (blue dot located at the posterior median) of cell-specific normalising constants ϕ_j (cellular mRNA content) and s_j (interpreted in terms of capture and reverse transcription efficiency for UMI counts), respectively. Panel (a) shows a substantial heterogeneity of the cells in terms of mRNA content. This is consistent with the evidence shown in [1], were the analysis suggested that the data contained samples from multiple distinct sub-populations. Moreover, panel (b) indicates that the scale of the technical counts remains relatively stable among cells.

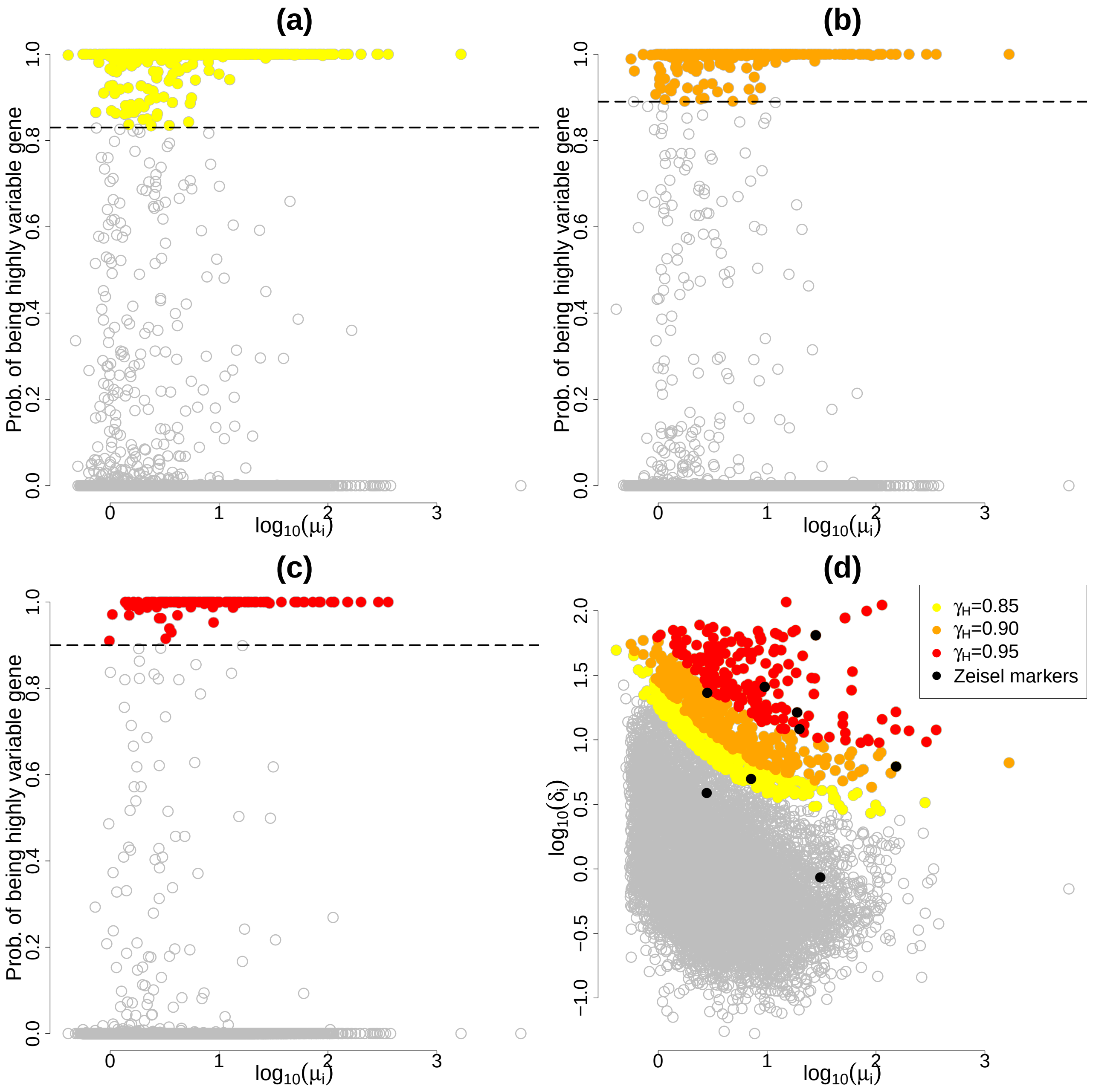

Figure S7: **Highly variable genes detection (Zeisel et al dataset).** (a), (b) and (c): for each of the 11,772 intrinsic genes, gene-specific expression rate μ_i (log scale) against the probability of being HVG for $\gamma_H =$ 85%, 90% and 95%, respectively. Black dashed lines located at optimal (i.e. when EFDR and EFNR coincide). Genes detected as highly variable are highlighted in yellow, orange and red, respectively. (d): for each of the 11,772 intrinsic genes, posterior medians of biological cell-to-cell heterogeneity terms δ_i (log scale) against posterior medians of expression levels μ_i (log scale). Genes detected as highly variable are highlighted in yellow, orange and red for variance contribution thresholds γ_H = 85%, 90% and 95%, respectively. Sub-population markers identified by [1] are highlighted in black. There is a good agreement between our list of highly variable genes and the list of sub-population markers identified by [1] (see Figure S7 (b)). In fact, for $\gamma_H = 80\%$, *Acta2* (99%), *Aif1* (95%), *Aldoc* (96%), *Cldn5* (98%), *Gad1* (97%), *Mbp* (93%) and *Spink8* (87%) belong to both groups (posterior medians of σ_i, the percentage of variance related to a biological cell-to-cell heterogeneity component, are shown in parenthesis). The two remaining sub-population markers highlighted by [1], *Tbr1* (76%) and *Thy1* (64%), are not classified as highly variable genes by our method. In particular, as shown Figure S8, most of the variability in expression counts that has been observed for *Thy1* disappears after removing the effect to cell-specific normalisation and unexplained technical variation.

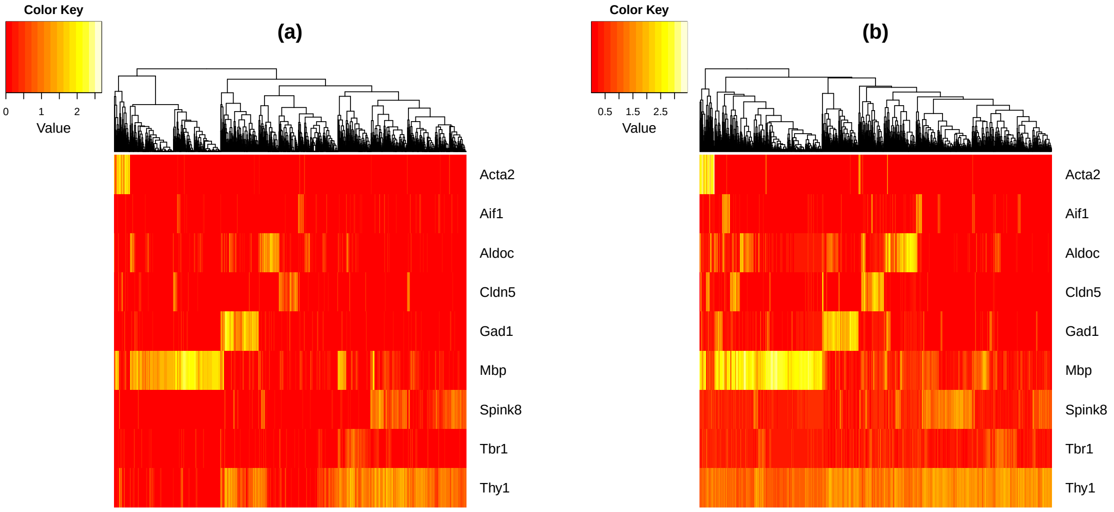

Figure S8: **Sub-population markers identified by [1].** Expression of *Acta2*, *Aif1*, *Aldoc*, *Cldn5*, *Gad1*, *Mbp*, *Spink8*, *Tbr1* and *Thy1*. (a): raw expression counts (log-scale, adding 1 pseudo-count) and (b): denoised expression rates where the effect of cell-specific normalisation and unexplained technical variation has been removed.
