# Supplementary material for: BASiCS: Bayesian Analysis of Single-Cell Sequencing Data
Source: PLoS Comput Biol. 2015 Jun 24;11(6):e1004333. doi: 10.1371/journal.pcbi.1004333 (PMC4480965; doi:10.1371/journal.pcbi.1004333)
Supplement: S1 Data — (ZIP) [file pcbi.1004333.s009.zip › AnalysisMouseESC/BASiCS_AnalysisMouseESC.html]

BASiCS analysis of Mouse ESC dataset


# BASiCS analysis of Mouse ESC dataset

#### *Catalina A. Vallejos, John C. Marioni and Sylvia Richardson*

#### *2015-05-27*

---

## Introduction

This document shows the code used when illustrating the use of BASiCS by analysing the mouse ESC dataset described in Islam et al (2014)1. To start the analysis, the following data must be dowloaded and stored in `data.path` directory.

- Expression counts. File ‘GSE46980\_CombinedMoleculeCounts.tab’ from Series GSE46980.
- Quality control information. File ‘187\_3lanes\_CA.txt’ (provided by Sten Linnarson).
- Input molecules of spike-in genes. File ‘SilverBulletCTRLConc.txt’ (provided by Sten Linnarson).
- List of highly variable genes detected by Islam et al (2014). Supplementary Table 1 (noisy genes) in Islam et al (2014)

```
# Here, we stored the 3 files mentioned above in the current R working directory
# Change this path as necessary

#setwd("/Users/catalinavallejos/Documents/MRC/Projects/SCE/LaTeX/BASiCS/AnalysisMouseESC/")
data.path = getwd()
```

In addition, the following libraries must be loaded before performing the analysis

```
#################################
# REQUIRED LIBRARIES ############
#################################
library(BASiCS) # To perform BASiCS analysis
```

```
## No methods found in "BiocGenerics" for requests: displayTechIndicator, displaySpikeInput
```

```
# Please ignore the message 'No methods found in "BiocGenerics" for requests: displayTechIndicator, displaySpikeInput'
# BASiCS only needs to import the generic functions, not the associated methods. 

#################################
# OPTIONAL LIBRARIES ############
#################################
library(data.table) # For fast pre-processing of large datasets. Can be replaced by standard 'data.frame' objects
```

```
## Warning: package 'data.table' was built under R version 3.1.3
```

```
library(DESeq) # To perform analysis according to the method described in Brennecke et al (2013)
```

```
## Loading required package: BiocGenerics
## Loading required package: parallel
## 
## Attaching package: 'BiocGenerics'
## 
## The following objects are masked from 'package:parallel':
## 
##     clusterApply, clusterApplyLB, clusterCall, clusterEvalQ,
##     clusterExport, clusterMap, parApply, parCapply, parLapply,
##     parLapplyLB, parRapply, parSapply, parSapplyLB
## 
## The following object is masked from 'package:stats':
## 
##     xtabs
## 
## The following objects are masked from 'package:base':
## 
##     anyDuplicated, append, as.data.frame, as.vector, cbind,
##     colnames, do.call, duplicated, eval, evalq, Filter, Find, get,
##     intersect, is.unsorted, lapply, Map, mapply, match, mget,
##     order, paste, pmax, pmax.int, pmin, pmin.int, Position, rank,
##     rbind, Reduce, rep.int, rownames, sapply, setdiff, sort,
##     table, tapply, union, unique, unlist
## 
## Loading required package: Biobase
## Welcome to Bioconductor
## 
##     Vignettes contain introductory material; view with
##     'browseVignettes()'. To cite Bioconductor, see
##     'citation("Biobase")', and for packages 'citation("pkgname")'.
## 
## Loading required package: locfit
## locfit 1.5-9.1    2013-03-22
## Loading required package: lattice
##     Welcome to 'DESeq'. For improved performance, usability and
##     functionality, please consider migrating to 'DESeq2'.
```

```
library(genefilter) # To use 'rowVars' function (Brennecke et al analysis)
```

```
## 
## Attaching package: 'genefilter'
## 
## The following object is masked from 'package:base':
## 
##     anyNA
```

```
library(statmod) # To use 'glmgam.fit' function (Brennecke et al analysis)
library(MCMCglmm) # To use 'posterior.mode' function. It requires an 'mcmc' object as input (required for some of the figures included in the main text)
```

```
## Loading required package: Matrix
## Loading required package: coda
```

```
## Warning: package 'coda' was built under R version 3.1.3
```

```
## Loading required package: ape
```

```
## Warning: package 'ape' was built under R version 3.1.2
```

```
library(coda) # To create 'mcmc' objects (required for some of the figures included in the main text)
library(xlsx) # To load the .xlsx file containing the list of highly variable genes provided by Islam et al (2014)
```

```
## Warning: package 'xlsx' was built under R version 3.1.1
```

```
## Loading required package: rJava
## Loading required package: xlsxjars
```

```
## Warning: package 'xlsxjars' was built under R version 3.1.1
```

---

## Data pre-processing

#### Loading the expression counts

```
# Matrix of expression counts (ignoring metadata)
Counts <- fread(paste0(data.path,"/GSE46980_CombinedMoleculeCounts.tab"),skip=6,drop=c("Chr","Pos","Strand","TrLen","MinExonHits","ExonHits"))
Cells <- as.vector(t(read.table(paste0(data.path,"/GSE46980_CombinedMoleculeCounts.tab"),skip=5, nrows = 1, header = F)[-1]))
SpikesInfo <- fread(paste0(data.path,"/SilverBulletCTRLConc.txt"), select=c("ERCC_ID","Name","molecules_in_each_chamber"))
```

- `Counts` is the matrix of expression counts. The first column in `Counts` contains the identifiers for each of the 25914 transcripts and the remaining columns contain the observed expression counts for each of the 96 cells in the sample.
- `Cells` contains cell identifiers for each of the 96 cells in the sample. These identifiers are required in order to combine the quality control information.
- `SpikesInfo` contains the input molecules that were added to the cell’s lysis (for each of the 92 spike-in genes)

The first step is to fix the format of genes and cells’ identifiers:

```
# Cell identifiers
setnames(Counts,names(Counts),c("Name",Cells)) 

# Gene identifiers 
# ERCC identifiers must start with 'ERCC' (required to combine with the information about input molecules)
setkey(Counts,Name); setkey(SpikesInfo,Name)
Counts <- merge(Counts,SpikesInfo,all=TRUE)
# Creating a variable containing gene names (including ERCC names)
Counts$Name <- ifelse(is.na(Counts$ERCC_ID),Counts$Name,Counts$ERCC_ID) 
# Excluding 4 spike-in genes that are not part of the ERCC molecules.
Counts <- Counts[-grep("SPIKE",Counts$Name)] 

# Removing a column which is no longer required
Counts=Counts[,ERCC_ID:=NULL]
```

**Current processed data contains 25910 genes and 96 cells.** The next step is to remove those cells that did not pass the quality control criteria employed by Islam et al (2014).

```
QC_Info <- fread(paste0(data.path,"/187_3lanes_CA.txt"))
GoodCells=QC_Info$Well[QC_Info$GoodCell==1]
# 9 other cells deleted as possible MEF (information provided by Sten Linnarson)
MEF=c("D02", "E02", "A06", "H07", "D08", "A09", "G10", "F12", "G12") 
GoodCells=GoodCells[!(GoodCells %in% MEF)]
Counts <- subset(Counts, select = c("Name",GoodCells,"molecules_in_each_chamber"))
```

**Current processed data contains 25910 genes and 41 cells.** The next step is to remove those transcrips that are very lowly expressed.

```
GenesIDs <- Counts$Name
# Fixing a gene name that was missinterpreted by excel... 
GenesIDs[GenesIDs=="1-Sep"]="Sept1" 
SpikesInput <- Counts$molecules_in_each_chamber
Counts = Counts[, Name := NULL]
Counts = Counts[, molecules_in_each_chamber := NULL]

SumByGene = rowSums(Counts)
GenesInclude = I(SumByGene>=41)
CountsQC = as.matrix(Counts[GenesInclude,]) 
GenesQC.IDs = GenesIDs[GenesInclude]
SpikesInputQC = SpikesInput[GenesInclude]
# Fixing a gene name that was missinterpreted by excel...
GenesQC.IDs[GenesQC.IDs=="1-Sep"]="Sept1" 
# Creating and identifier of spike-in genes
TechQC=ifelse(1:nrow(CountsQC) %in% grep("ERCC",GenesQC.IDs),T,F)
```

**Current processed data contains 7941 genes and 41 cells.** Finally, we need to re-arrange the data such that the expression counts are at the bottom of the table.

```
CountsQC=rbind(CountsQC[!TechQC,],CountsQC[TechQC,])
GenesQC.IDs=c(GenesQC.IDs[!TechQC],GenesQC.IDs[TechQC])
SpikesInputQC = c(SpikesInputQC[!TechQC],SpikesInputQC[TechQC]) 
TechQC=c(TechQC[!TechQC],TechQC[TechQC]) 
SpikesInputQC = SpikesInputQC[TechQC]

n = ncol(CountsQC) # Number of cells
q = nrow(CountsQC) # Total number of genes
q.bio = q - sum(TechQC) # Number of intrinsic genes
```

**Final processed data contains 7941 genes (7895 biological and 46 spike-in) and 41 cells.**

---

## Analysis according to the method in Brennecke et al (2013)

```
# Normalisation (separately for technical and biological genes)
sfTec <- estimateSizeFactorsForMatrix( CountsQC[TechQC,] ) 
sfBio <- estimateSizeFactorsForMatrix( CountsQC[!TechQC,] )
nCountsTec <- t( t(CountsQC[TechQC,]) / sfTec )
nCountsBio <- t( t(CountsQC[!TechQC,]) / sfBio )

# Estimation of gene-specific expression rates
meansTec <- rowMeans( nCountsTec ); varsTec <- rowVars( nCountsTec ); cv2Tec <- varsTec / meansTec^2
meansBio <- rowMeans( nCountsBio ); varsBio <- rowVars( nCountsBio ); cv2Bio <- varsBio / meansBio^2

# HVG detection
minMeanForFit <- unname( quantile( meansTec[ which( cv2Tec > .3 ) ], .95 ) )
useForFit <- meansTec >= minMeanForFit
fit <- glmgam.fit( cbind( a0 = 1, a1tilde = 1/meansTec[useForFit] ),cv2Tec[useForFit] )
xi <- mean( 1 / sfTec )
a0 <- unname( fit$coefficients["a0"] )
a1 <- unname( fit$coefficients["a1tilde"] - xi )
psia1theta <- mean( 1 / sfBio ) + a1 * mean( sfTec / sfBio )
minBiolDisp <- .5^2
m <- ncol(CountsQC[TechQC==F,])
cv2th <- a0 + minBiolDisp + a0 * minBiolDisp
testDenom <- ( meansBio * psia1theta + meansBio^2 * cv2th ) / ( 1 + cv2th/m )
p <- 1 - pchisq( varsBio * (m-1) / testDenom, m-1 )
padj <- p.adjust( p, "BH" )
sig <- padj < .1
sig[is.na(sig)] <- FALSE
HVG_Bren_id=which(sig)
```

Using this method, 1363 intrinsic genes are classified as highly variable.

---

## List of highly variable genes provided by Islam et al (2014)

```
IslamInfo <- read.xlsx(paste0(data.path,"/nmeth.2772-S2.xlsx"), 1 , stringsAsFactors=F)
IslamInfo[! IslamInfo$Gene %in% GenesQC.IDs,] # Genes excluded from our analysis becase of low total counts.
```

```
##            Gene    Mean  CV
## 2 1700001L05Rik 1.87192 356
## 6          Aass 1.92073 366
## 9         Ap1s3 2.22573 280
```

```
HVG_Islam_id=which(GenesQC.IDs %in% IslamInfo$Gene)
```

Using this method, 115 intrinsic genes are classified as highly variable.

## BASiCS analysis

#### The input dataset

To use BASiCS, we need to create a `BASiCS_Data` object containing the expression counts, a vector of spike-in gene indicators (`TRUE/FALSE`) and the input number of mRNA molecules for each spike-in gene.

```
Data = newBASiCS_Data(CountsQC, TechQC, SpikesInputQC)
```

```
## An object of class BASiCS_Data
##  Dataset contains 7941 genes (7895 biological and 46 technical) and 41 cells.
##  Elements (slots): Counts, Tech and SpikeInput.
## 
## NOTICE: BASiCS requires a pre-filtered dataset 
##     - You must remove poor quality cells before creating the BASiCS data object 
##     - We recommend to pre-filter very lowly expressed transcripts before creating the object. 
##       Inclusion criteria may vary for each data. For example, remove transcripts 
##           - with very low total counts across of all samples 
##           - that are only expressed in few cells 
##             (by default genes expressed in only 1 cell are not accepted) 
##           - with very low total counts across the samples where the transcript is expressed 
## 
##  BASiCS_Filter can be used for this purpose
```

#### Fitting the BASiCS model

To run the MCMC algorithm, we use the function `BASiCS_MCMC`. Here, we set all hyper-parameters equal to 1.

```
#MCMC_Output = BASiCS_MCMC(Data, N = 20000, Thin = 10, Burn = 10000, PrintProgress = F)
MCMC_Output = BASiCS_MCMC(Data, N = 20000, Thin = 10, Burn = 10000, PrintProgress = F)
```

```
## --------------------------------------------------------------------
## MCMC sampler has been started: 20000 iterations to go.
## --------------------------------------------------------------------
## --------------------------------------------------------------------
## End of burn-in period.
## --------------------------------------------------------------------
##  
## --------------------------------------------------------------------
## --------------------------------------------------------------------
## All 20000 MCMC iterations have been completed.
## --------------------------------------------------------------------
## --------------------------------------------------------------------
##  
## --------------------------------------------------------------------
## Please see below a summary of the overall acceptance rates.
## --------------------------------------------------------------------
##  
## Minimum acceptance rate among mu[i]'s: 0.3748
## Average acceptance rate among mu[i]'s: 0.51481
## Maximum acceptance rate among mu[i]'s: 0.7274
##  
## Minimum acceptance rate among delta[i]'s: 0.4415
## Average acceptance rate among delta[i]'s: 0.534844
## Average acceptance rate among delta[i]'s: 0.8026
##  
## Minimum acceptance rate among kappa[j]'s: 0.4332
## Average acceptance rate among kappa[j]'s: 0.452027
## Maximum acceptance rate among kappa[j]'s: 0.471
##  
## Minimum acceptance rate among nu[j]'s: 0.4169
## Average acceptance rate among nu[j]'s: 0.44492
## Maximum acceptance rate among nu[j]'s: 0.4595
##  
## Acceptance rate for theta: 0.8059
## --------------------------------------------------------------------
##  
## --------------------------------------------------------------------
## MCMC running time
## --------------------------------------------------------------------
##     user   system  elapsed 
## 2853.933   95.625 7456.093 
## 
## --------------------------------------------------------------------
## Output
## --------------------------------------------------------------------
## An object of class BASiCS_Chain
##  1000 MCMC samples.
##  Dataset contains 7941 genes (7895 biological and 46 technical) and 41 cells.
##  Elements (slots): mu, delta, phi, s, nu and theta.
```

To assess convergence of the chain, the convergence diagnostics provided by the package `coda` can be used. Additionally, a visual inspection is provided by traceplots, for example:

```
par(mgp=c(5,1,0)); par(mar=c(7,9,4,0.5)); par(mfrow=c(3,2))
plot(MCMC_Output, Param = "mu", Gene = 1, cex.lab = 2)
plot(MCMC_Output, Param = "delta", Gene = 1, cex.lab = 2)
plot(MCMC_Output, Param = "phi", Cell = 1, cex.lab = 2)
plot(MCMC_Output, Param = "s", Cell = 1, cex.lab = 2)
plot(MCMC_Output, Param = "nu", Cell = 1, cex.lab = 2)
plot(MCMC_Output, Param = "theta", cex.lab = 2)
```

#### Summarizing the model fit

To summarize the results, the function `Summary` calculates posterior medians and the High Posterior Density (HPD) intervals for each model parameter. As a default option, HPD intervals contain 0.95 probability.

```
MCMC_Summary <- Summary(MCMC_Output)
head(displaySummaryMu(MCMC_Summary))
```

```
##             Mu     lower     upper
## var1  8.202876  5.139092 11.905359
## var2 10.667867  7.068047 14.456868
## var3  4.942627  2.955094  7.630980
## var4  6.144896  3.693061  9.256696
## var5 21.681496 16.290014 27.936334
## var6 12.838129  9.875983 16.443286
```

```
head(displaySummaryDelta(MCMC_Summary))
```

```
##          Delta     lower     upper
## var1 1.2357233 0.5687319 2.1534997
## var2 0.8934631 0.3701011 1.5468271
## var3 1.5197111 0.6129799 2.6846489
## var4 1.5245022 0.7231801 2.7465162
## var5 0.5316314 0.2601823 0.9470649
## var6 0.4084082 0.1619650 0.7947863
```

```
head(displaySummaryPhi(MCMC_Summary))
```

```
##           Phi     lower     upper
## var1 1.068651 1.0232189 1.1248908
## var2 1.132755 1.1004276 1.1613009
## var3 1.165209 1.1327453 1.1966068
## var4 1.120181 1.0919895 1.1512169
## var5 0.851961 0.8286403 0.8773731
## var6 1.075790 1.0482842 1.1064535
```

```
head(displaySummaryS(MCMC_Summary))
```

```
##              S      lower    upper
## var1 0.3812280 0.07777273 1.281444
## var2 0.4116489 0.07578549 1.342358
## var3 0.3877426 0.07452562 1.333508
## var4 0.3975925 0.08744929 1.383250
## var5 0.3756211 0.06693769 1.268793
## var6 0.3955919 0.07779230 1.309991
```

```
head(displaySummaryNu(MCMC_Summary))
```

```
##             Nu     lower     upper
## var1 0.2654422 0.2555881 0.2735322
## var2 0.2931209 0.2865417 0.2989842
## var3 0.2740734 0.2676038 0.2793736
## var4 0.2759257 0.2694894 0.2820111
## var5 0.2505074 0.2447978 0.2563018
## var6 0.2789405 0.2731178 0.2846139
```

```
head(displaySummaryTheta(MCMC_Summary))
```

```
##          Theta     lower     upper
## var1 0.4140722 0.1457055 0.8021185
```

#### Manuscript’s Figure 4 - Normalisation

```
par(mgp=c(5,1,0)); par(mar=c(7,9,4,0.5)); par(mfrow=c(2,2))
aux=sfBio/sfTec; 
plot(aux,displaySummaryPhi(MCMC_Summary)[,1],ylim=c(0.35,1.6),xlim=c(0.35,1.6),cex=1,pch=16,col="blue",bty="n",cex.main=1,cex.lab=1,cex.axis=1, xlab="Brennecke et al (2013)",ylab="BASiCS",main="(a)")
abline(a=0,b=1,col=8,lty=2,lwd=3)
for(cell in 1:n) 
{
  segments(x0=aux[cell],y0=displaySummaryPhi(MCMC_Summary)[cell,2],y1=displaySummaryPhi(MCMC_Summary)[cell,3],col="blue",lwd=3.5)
  segments(x0=aux[cell]-0.0125,y0=displaySummaryPhi(MCMC_Summary)[cell,2],x1=aux[cell]+0.0125,col="blue",lwd=2)
  segments(x0=aux[cell]-0.0125,y0=displaySummaryPhi(MCMC_Summary)[cell,3],x1=aux[cell]+0.0125,col="blue",lwd=2)
}
plot(MCMC_Summary, Param = "s", main = "(b)", cex=2.5)
points(1:n,sfTec,col="red",cex=2.5,pch=16)
points(1:n,colSums(CountsQC[TechQC==T,])/sum(SpikesInputQC),col="black",cex=2.5,pch=16)

hist(displayChainS(MCMC_Output)[,1],breaks=25,main="(c)",ylab="Frequency",xlab=expression(s[1]),cex.main=1,cex.lab=1,cex.axis=1,col=8)
abline(v=displaySummaryS(MCMC_Summary)[1,1],col="red",lwd=3)
abline(v=posterior.mode(mcmc(displayChainS(MCMC_Output)[,1])),col="blue",lwd=3)
legend('topright',c(expression(paste("Post. median of ",s[1])),"Capture prop. (cell 1)"),lty=1,bty="n",cex=1,lwd=3,col=c("red","blue"))

hist(displayChainS(MCMC_Output)[,2],breaks=25,main="(d)",ylab="Frequency",xlab=expression(s[2]),cex.main=1,cex.lab=1,cex.axis=1,col=8)
abline(v=displaySummaryS(MCMC_Summary)[2,1],col="red",lwd=3)
abline(v=posterior.mode(mcmc(displayChainS(MCMC_Output)[,2])),col="blue",lwd=3)
legend('topright',c(expression(paste("Post. median of ",s[2])),"Capture prop. (cell 2)"),lty=1,bty="n",cex=1,lwd=3,col=c("red","blue"))
```

#### Manuscript’s Figure 5 - Technical variability

```
par(mar=c(5,6,4,2)); par(mfrow=c(1,2))
plot(MCMC_Summary, Param = "nu", main = "(a)", cex = 2.5, lwd = 3.5)
hist(displayChainTheta(MCMC_Output),breaks=25,main="(b)",ylab="Frequency",xlab=expression(theta),cex.main=1,cex.lab=1,cex.axis=1,col=8)
```

#### Manuscript’s Figure 6 - Highly and lowly variable genes detection

If there is no clear pre-determined choice for the evidence thresholds \(\gamma\_H\) and \(\gamma\_L\), a grid search can be used in order to determine those values for which EFDR and EFNR coincide and are equal to a pre-specified error rate (e.g. EFDR = EFNR = 10%)

```
# Grid search for variance contribution thresholds such that EFDR = EFNR = 10%
BASiCS_VarThresholdSearchHVG(MCMC_Output, VarThresholdsGrid = seq(0.75,0.80,by=0.01))
```

```
## 549  genes classified as highly variable using: 
## - Variance contribution threshold =  75 % 
## - Evidence threshold =  0.7255 
## - EFDR =  14.12 % 
## - EFNR =  13.87 % 
## 396  genes classified as highly variable using: 
## - Variance contribution threshold =  76 % 
## - Evidence threshold =  0.7515 
## - EFDR =  13.02 % 
## - EFNR =  13 % 
## 277  genes classified as highly variable using: 
## - Variance contribution threshold =  77 % 
## - Evidence threshold =  0.7705 
## - EFDR =  12.06 % 
## - EFNR =  11.97 % 
## 188  genes classified as highly variable using: 
## - Variance contribution threshold =  78 % 
## - Evidence threshold =  0.7815 
## - EFDR =  10.87 % 
## - EFNR =  10.8 % 
## 126  genes classified as highly variable using: 
## - Variance contribution threshold =  79 % 
## - Evidence threshold =  0.801 
## - EFDR =  9.56 % 
## - EFNR =  9.53 % 
## 81  genes classified as highly variable using: 
## - Variance contribution threshold =  80 % 
## - Evidence threshold =  0.8255 
## - EFDR =  8.34 % 
## - EFNR =  8.26 %
```

```
##      Var. Threshold (%) EFDR (%) EFNR (%) Optimal evidence thres.
## [1,]                 75    14.12    13.87                  0.7255
## [2,]                 76    13.02    13.00                  0.7515
## [3,]                 77    12.06    11.97                  0.7705
## [4,]                 78    10.87    10.80                  0.7815
## [5,]                 79     9.56     9.53                  0.8010
## [6,]                 80     8.34     8.26                  0.8255
##      # Detected genes
## [1,]              549
## [2,]              396
## [3,]              277
## [4,]              188
## [5,]              126
## [6,]               81
```

```
BASiCS_VarThresholdSearchLVG(MCMC_Output, VarThresholdsGrid = seq(0.40,0.45,by=0.01))
```

```
## 640  genes classified as lowly variable using: 
## - Variance contribution threshold =  40 % 
## - Evidence threshold =  0.7465 
## - EFDR =  8.94 % 
## - EFNR =  8.93 % 
## 707  genes classified as lowly variable using: 
## - Variance contribution threshold =  41 % 
## - Evidence threshold =  0.7355 
## - EFDR =  9.31 % 
## - EFNR =  9.3 % 
## 781  genes classified as lowly variable using: 
## - Variance contribution threshold =  42 % 
## - Evidence threshold =  0.7205 
## - EFDR =  9.72 % 
## - EFNR =  9.67 % 
## 860  genes classified as lowly variable using: 
## - Variance contribution threshold =  43 % 
## - Evidence threshold =  0.7095 
## - EFDR =  10.1 % 
## - EFNR =  10.05 % 
## 944  genes classified as lowly variable using: 
## - Variance contribution threshold =  44 % 
## - Evidence threshold =  0.6995 
## - EFDR =  10.46 % 
## - EFNR =  10.44 % 
## 1037  genes classified as lowly variable using: 
## - Variance contribution threshold =  45 % 
## - Evidence threshold =  0.6955 
## - EFDR =  10.84 % 
## - EFNR =  10.8 %
```

```
##      Var. Threshold (%) EFDR (%) EFNR (%) Optimal evidence thres.
## [1,]                 40     8.94     8.93                  0.7465
## [2,]                 41     9.31     9.30                  0.7355
## [3,]                 42     9.72     9.67                  0.7205
## [4,]                 43    10.10    10.05                  0.7095
## [5,]                 44    10.46    10.44                  0.6995
## [6,]                 45    10.84    10.80                  0.6955
##      # Detected genes
## [1,]              640
## [2,]              707
## [3,]              781
## [4,]              860
## [5,]              944
## [6,]             1037
```

Highly and lowly variable genes detection using the same thresholds as in the paper:

```
DetectHVG <- BASiCS_DetectHVG(MCMC_Output, VarThreshold = 0.79, EviThreshold = 0.7925)
```

```
## 132  genes classified as highly variable using: 
## - Variance contribution threshold =  79 % 
## - Evidence threshold =  0.7925 
## - EFDR =  10.04 % 
## - EFNR =  9.47 %
```

```
DetectLVG <- BASiCS_DetectLVG(MCMC_Output, VarThreshold = 0.41, EviThreshold = 0.7650)
```

```
## 657  genes classified as lowly variable using: 
## - Variance contribution threshold =  41 % 
## - Evidence threshold =  0.765 
## - EFDR =  8.11 % 
## - EFNR =  9.76 %
```

For these thresholds, BASiCS detects 0 highly variable genes and 0 lowly variable genes.

```
par(mar=c(5,6,4,0.5)); par(mfrow=c(1,2))
hist(DetectHVG$Table[,4],col=8,cex=2,lwd=2,
     xlab="Overall biological cell-to-cell heterogeneity contribution",main="(b)",cex.main=1,cex.lab=0.9,cex.axis=1,bty="n")
abline(v=median(DetectHVG$Table[,4]),col="red",lwd=3)
legend('topleft',"Median value",lty=1,bty="n",cex=1,lwd=3,col="red")

BASiCS_ContoursDelta<-function(
  Mu=exp(seq(0.5,12,by=0.1)), # Grid of values for gene-specific expression rates.  
  VarThreshold, # Variance contribution threshold. Value must be between 0 and 1. Use large values if HVG=T, use small values if HVG=F
  PhiS, # Vector of length $n$ whose elements correspond to the product of the corresponding posterior medians of $\phi_j$ and $s_j$   
  Theta) # Posterior median of $\theta$
{  
  return((VarThreshold/(1-VarThreshold))*((Theta+(median(PhiS)*Mu)^(-1) )/(Theta+1)))  
}

plot(MCMC_Summary, Param = "mu", Param2 = "delta", main = "(b)", log="xy", col = 8)
mu=10^seq(0.5,12,by=0.1)
lines(mu,BASiCS_ContoursDelta(Mu=mu,VarThreshold=0.60,PhiS=displaySummaryPhi(MCMC_Summary)[,1]*displaySummaryS(MCMC_Summary)[,1],Theta=displaySummaryTheta(MCMC_Summary)[,1]),col="red",lwd=3,lty=2)
lines(mu,BASiCS_ContoursDelta(Mu=mu,VarThreshold=0.70,PhiS=displaySummaryPhi(MCMC_Summary)[,1]*displaySummaryS(MCMC_Summary)[,1],Theta=displaySummaryTheta(MCMC_Summary)[,1]),col="red",lwd=3,lty=1)
lines(mu,BASiCS_ContoursDelta(Mu=mu,VarThreshold=0.30,PhiS=displaySummaryPhi(MCMC_Summary)[,1]*displaySummaryS(MCMC_Summary)[,1],Theta=displaySummaryTheta(MCMC_Summary)[,1]),col="blue",lwd=3,lty=1)
lines(mu,BASiCS_ContoursDelta(Mu=mu,VarThreshold=0.20,PhiS=displaySummaryPhi(MCMC_Summary)[,1]*displaySummaryS(MCMC_Summary)[,1],Theta=displaySummaryTheta(MCMC_Summary)[,1]),col="blue",lwd=3,lty=2)
legend('topright',legend=c(expression(paste(gamma[L],"=0.20")),expression(paste(gamma[L],"=0.30")),expression(paste(gamma[H],"=0.60")),expression(paste(gamma[H],"=0.70"))), col=c("blue","blue","red","red"),lty=c(2,1,2,1),bty="n",cex=1,lwd=3)
```

#### Manuscript’s Figure 7

```
par(mar=c(5,6,4,0.5)); par(mfrow=c(1,2))
plot(DetectHVG$Table[,2],DetectHVG$Table[,5],pch=1,cex=1.5,lwd=2,col=8, log = "x",
     ylab="Prob. of being highly variable gene",xlab=expression(log[10](mu[i])), main="(a)",cex.main=1,cex.lab=1,cex.axis=1,bty="n")
points(DetectHVG$Table[which(DetectHVG$Table[,5]>0.7925),2],DetectHVG$Table[which(DetectHVG$Table[,5]>0.7925),5],col="red",pch=16,cex=1.5)
abline(h=0.7925, lty=5, col="black", lwd=3)

plot(DetectLVG$Table[,2],DetectLVG$Table[,5],pch=1,cex=1.5,lwd=2,col=8, log = "x",
     ylab="Prob. of being lowly variable gene",xlab=expression(log[10](mu[i])), main="(b)",cex.main=1,cex.lab=1,cex.axis=1,bty="n")
points(DetectLVG$Table[which(DetectLVG$Table[,5]>0.7650),2],DetectLVG$Table[which(DetectLVG$Table[,5]>0.7650),5],col="blue",pch=16,cex=1.5)
abline(h=0.7650, lty=5, col="black", lwd=3)
```

#### Manuscript’s Figure 8

```
par(mgp=c(6,2,0)); par(mar=c(8,10,4,0.5)); par(mfrow=c(1,3))
plot(MCMC_Summary, Param = "mu", Param2 = "delta", main = "(a)", log="xy", cex.main = 2, cex.lab = 2, cex.axis = 1.5, col = 8, cex = 1.5)
points(DetectHVG$Table[which(DetectHVG$Table[,5]>0.7925),2],DetectHVG$Table[which(DetectHVG$Table[,5]>0.7925),3],cex=1.5,pch=16,col="red")
points(DetectLVG$Table[which(DetectLVG$Table[,5]>0.7650),2],DetectLVG$Table[which(DetectLVG$Table[,5]>0.7650),3],cex=1.5,pch=16,col="blue")
legend('topright',c("HVG (BASiCS)","LVG (BASiCS)"),col=c("red","blue"),pch=16,cex=2,bty="n")

plot(MCMC_Summary, Param = "mu", Param2 = "delta", main = "(b)", log="xy", cex.main = 2, cex.lab = 2, cex.axis = 1.5, col = 8, cex = 1.5)
points(displaySummaryMu(MCMC_Summary)[1:q.bio,1][HVG_Islam_id],displaySummaryDelta(MCMC_Summary)[1:q.bio,1][HVG_Islam_id],cex=1.5,pch=16,col="red")
legend('topright',c("HVG (Islam et al)"),col=c("red"),pch=16,cex=2,bty="n")

plot(MCMC_Summary, Param = "mu", Param2 = "delta", main = "(c)", log="xy", cex.main = 2, cex.lab = 2, cex.axis = 1.5, col = 8, cex = 1.5)
points(displaySummaryMu(MCMC_Summary)[1:q.bio,1][HVG_Bren_id],displaySummaryDelta(MCMC_Summary)[1:q.bio,1][HVG_Bren_id],cex=1.5,pch=16,col="red")
legend('topright',c("HVG (Brennecke et al)"),col=c("red"),pch=16,cex=2,bty="n")
```

---

## R session information

```
sessionInfo()
```

```
## R version 3.1.0 (2014-04-10)
## Platform: x86_64-apple-darwin10.8.0 (64-bit)
## 
## locale:
## [1] en_GB.UTF-8/en_GB.UTF-8/en_GB.UTF-8/C/en_GB.UTF-8/en_GB.UTF-8
## 
## attached base packages:
## [1] parallel  stats     graphics  grDevices utils     datasets  methods  
## [8] base     
## 
## other attached packages:
##  [1] xlsx_0.5.7          xlsxjars_0.6.1      rJava_0.9-6        
##  [4] MCMCglmm_2.21       ape_3.2             coda_0.17-1        
##  [7] Matrix_1.1-4        statmod_1.4.20      genefilter_1.46.1  
## [10] DESeq_1.16.0        lattice_0.20-29     locfit_1.5-9.1     
## [13] Biobase_2.24.0      BiocGenerics_0.10.0 data.table_1.9.4   
## [16] BASiCS_0.1.4       
## 
## loaded via a namespace (and not attached):
##  [1] annotate_1.42.1      AnnotationDbi_1.26.1 chron_2.3-45        
##  [4] corpcor_1.6.7        DBI_0.3.1            digest_0.6.4        
##  [7] evaluate_0.5.5       formatR_1.0          geneplotter_1.42.0  
## [10] GenomeInfoDb_1.0.2   grid_3.1.0           htmltools_0.2.6     
## [13] IRanges_1.22.10      knitr_1.9            nlme_3.1-118        
## [16] plyr_1.8.1           RColorBrewer_1.0-5   Rcpp_0.11.6         
## [19] reshape2_1.4         rmarkdown_0.5.1      RSQLite_1.0.0       
## [22] splines_3.1.0        stats4_3.1.0         stringr_0.6.2       
## [25] survival_2.37-7      tensorA_0.36         tools_3.1.0         
## [28] XML_3.98-1.1         xtable_1.7-4         yaml_2.1.13
```

---

1. Islam et al (2014). Quantitative single-cell RNA-seq with unique molecular identifiers. *Nature Methods* 11: 163-166.↩
